# A cluster randomized trial of a multifaceted quality improvement intervention in Brazilian intensive care units: study protocol

**DOI:** 10.1186/s13012-014-0190-0

**Published:** 2015-01-13

**Authors:** Alexandre Biasi Cavalcanti, The CHECKLIST-ICU Investigators and the BRICNet

**Affiliations:** Research Institute - Hospital do Coração (IEP– HCor), Rua Abílio Soares 250, 12th floor, CEP: 04005-000 - São Paulo SP, Brazil

**Keywords:** Intensive care, Critical illness, Intensive care units, Checklist, Hospital mortality, Outcome and process assessment (health care), Quality improvement

## Abstract

**Background:**

The uptake of evidence-based therapies in the intensive care environment is suboptimal, particularly in limited-resource countries. Checklists, daily goal assessments, and clinician prompts may improve compliance with best practice processes of care and, in turn, improve clinical outcomes. However, the available evidence on the effectiveness of checklists is unreliable and inconclusive, and the mechanisms are poorly understood. We aim to evaluate whether the use of a multifaceted quality improvement intervention, including the use of a checklist and the definition of daily care goals during multidisciplinary daily rounds and clinician prompts, can improve the in-hospital mortality of patients admitted to intensive care units (ICUs). Our secondary objectives are to assess the effects of the study intervention on specific processes of care, clinical outcomes, and the safety culture and to determine which factors (the processes of care and/or safety culture) mediate the effect of the study intervention on mortality.

**Methods/design:**

This is a cluster randomized trial involving 118 ICUs in Brazil conducted in two phases. In the observational preparatory phase, we collect baseline data on processes of care and clinical outcomes from 60 consecutive patients with lengths of ICU stay longer than 48 h and apply the Safety Attitudes Questionnaire (SAQ) to 75% or more of the health care staff in each ICU. In the randomized phase, we assign ICUs to the experimental or control arm and repeat data collection. Experimental arm ICUs receive the multifaceted quality improvement intervention, including a checklist and definition of daily care goals during daily multidisciplinary rounds, clinician prompting, and feedback on rates of adherence to selected care processes. Control arm ICUs maintain usual care. The primary outcome is in-hospital mortality, truncated at 60 days. Secondary outcomes include the rates of adherence to appropriate care processes, rates of other clinical outcomes, and scores on the SAQ domains. Analysis follows the intention-to-treat principle, and the primary outcome is analyzed using mixed effects logistic regression.

**Discussion:**

This is a large scale, pragmatic cluster-randomized trial evaluating whether a multifaceted quality improvement intervention, including checklists applied during the multidisciplinary daily rounds and clinician prompting, can improve the adoption of proven therapies and decrease the mortality of critically ill patients. If this study finds that the intervention reduces mortality, it may be widely adopted in intensive care units, even those in limited-resource settings.

**Trial registration:**

ClinicalTrials.gov NCT01785966

**Electronic supplementary material:**

The online version of this article (doi:10.1186/s13012-014-0190-0) contains supplementary material, which is available to authorized users.

Modern intensive care requires a sophisticated, well-coordinated delivery system consisting of both advanced technology and a well-integrated and highly skilled team. Although significant advances have improved the care and outcomes of many critically ill patients, the complexity and stress of the intensive care unit (ICU) nonetheless predispose these units to considerable medical error. In particular, failure to implement the best evidence-based interventions in the ICU has been estimated to cause 160,000 avoidable deaths each year in the US [1]. For instance, use of low tidal volume ventilation was shown in a large NIH-funded study to reduce mortality in acute respiratory distress syndrome patients by 25%, yet many ICUs were still failing to implement this strategy many years after the study was published [2]. Similar evidence exists regarding the implementation of best practices for the care of sepsis patients [3]. Unfortunately, studies of ICU practices in developing countries suggest that compliance with best practices is worse than that reported in developed countries [4,5], with recent calls for greater interest to be taken in quality improvement as a global health priority [6].

Checklists have been successfully employed in aviation and the manufacturing industry to avoid critical omissions during complex procedures [7]. Furthermore, as they are typically read out by someone other than the team leader, they are key to make all members of the team speak up and, in so doing, promote a flatter hierarchy [8]. More recently, checklists have been successfully used in health care. Notable examples are the World Health Organization Checklist for Safe Surgery [9] and the Keystone ICU Project checklist to prevent central line-associated bloodstream infections [10]. Checklists have also been used during daily multidisciplinary ICU rounds to avoid errors of omission [11] and, together with daily goals assessment, may improve the effectiveness of communication [12]. In addition, the effectiveness of the checklists themselves can be leveraged by systematically prompting physicians to address omitted items [13].

Despite these successful examples, important concerns persist. First, the studies themselves often relied on simple “before-and-after” designs. Not surprisingly, conflicting results have been reported [14,15]. Second, little information was provided on which specific elements of checklist implementation were key to success. Indeed, the reported benefits sometimes appeared to exceed those that could be plausibly explained by the improvement in the specific processes targeted by the checklist.

We hypothesize that checklists and clinician prompting decrease mortality in ICU patients. We also believe they work not only through direct changes in processes of care but also by promoting a safer culture with flattened hierarchy, when all voices contribute to make sure nothing is missed, and thereby avoiding over-reliance on the potentially flawed and inconsistent mind and decisions of the team-leader, someone who being human can make mistakes [16].

## Objectives

We propose a cluster randomized trial to assess whether the use of a multifaceted quality improvement intervention, including checklists and definition of daily care goals during multidisciplinary rounds, as well as clinician prompting, can improve the in-hospital mortality of patients admitted to ICUs. Our secondary objective is to assess the effects of the study intervention on care processes, clinical outcomes, and safety culture.

Furthermore, we want to better characterize the mechanisms that mediate improvements in clinical outcomes, that is, whether a potential clinical benefit is mediated only through improved compliance with the processes targeted by the checklist or also through flattening of hierarchy and promotion of greater solicitation of input from the entire ICU team.

## Methods

### Study design

**Figure 1:**
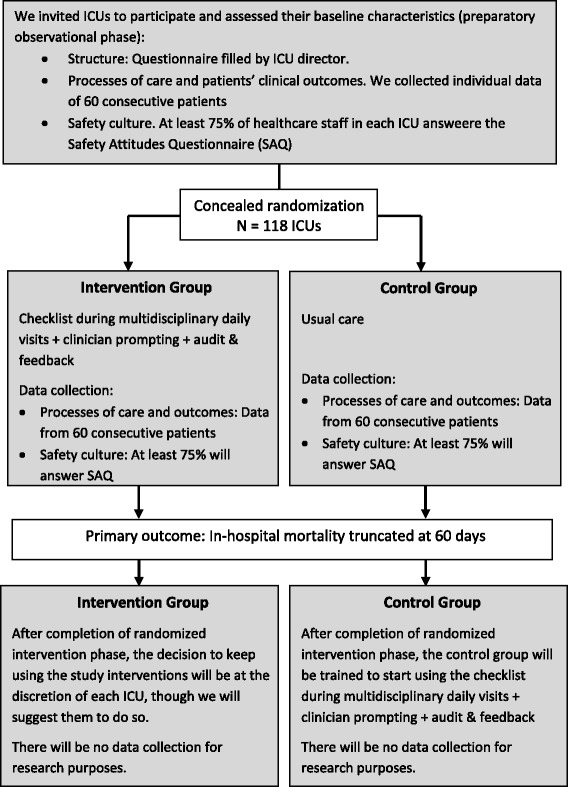
**Study design.**

This is a pragmatic two-arm cluster randomized trial involving ICUs in Brazil to determine the effectiveness of a multifaceted quality improvement intervention to reduce in-hospital mortality conducted in two phases (Figure 1). In the observational preparatory phase, we collect baseline data to characterize our sample, obtain outcome data for the stratified randomization and for adjusting multivariate analyses for baseline rates of clinical outcomes. In the next phase, we randomize ICUs to the experimental or control arm. The unit of concealed randomization is the ICU to minimize contamination, as we intend to apply the intervention to the whole ICU multidisciplinary team. Analysis is performed according to the intention-to-treat principle and accounts for the cluster randomized design. The study protocol is registered at www.ClinicalTrials.gov (NCT01785966) and is in accordance with the CONSORT 2010 Statement: Extension to cluster randomized trials (Additional file 1).

### Participants

#### Cluster eligibility criteria and recruitment

We include ICUs that primarily admit adult patients and conduct (or want to conduct) multidisciplinary daily rounds with at least a physician and a nurse on all working days. ICUs that admit exclusively cardiac patients, step down units, and ICUs that already systematically use multiple-item checklists during multidisciplinary daily visits are excluded. We define the systematic use of checklists as instances when a structured assessment (according to a printed or digital document) of multiple items focused on the prevention of usual ICU complications and/or when the explicit definition of daily goals is in use at least 3 days a week for more than 30 days, with or without a written record. For the randomized phase, we include only ICUs that successfully collect data in the observational phase (include ≥40 patients within 6 months and apply the Safety Attitudes Questionnaire to ≥75% of their staff).

We invited all members of the Brazilian Research in Intensive Care Network (BRICNet), the Associação Brasileira de Medicina Intensiva—AMIB (Brazilian Association of Intensive Care) and AMIB-Net to participate in the trial.

#### Patient eligibility criteria and recruitment

We include 60 consecutive patients over 18 years old with lengths of ICU stay longer than 48 h in each ICU; patients with lengths of stay less than 48 h are unlikely to be affected by the study interventions. We exclude patients with high probabilities of early death (defined as death occurring between the 48th and 72nd hour of ICU stay), patients admitted only for palliative care, and patients with a suspected or confirmed diagnosis of brain death.

### Interventions

#### Experimental arm

ICUs randomized to the experimental arm receive a multifaceted intervention comprising checklists and daily goals definition during daily multidisciplinary visits, as well as clinician prompting. The intervention is applicable to all patients during their whole ICU stay.

#### Checklist

The daily rounds checklist was developed following the five steps outlined below, adapted from the Clinical Practice Guideline Development Cycle, a transparent process for the development of evidence-based guidelines.

In step 1, the members of the Steering Committee listed some items that should potentially be included in the checklist based on the existing literature. The following items were considered: 1) venous thromboembolism prophylaxis; 2) screening for severe sepsis; 3) adjustment/discontinuation of antibiotics; 4) removal of venous central line; 5) removal of indwelling urinary catheter; 6) elevation of the bed head at 30° or more; 7) pain control; 8) light sedation; 9) discontinuation of mechanical ventilation; 10) tidal volume control; 11) oral hygiene with chlorhexidine; and 12) achieving optimal individual nutritional requirements.

In step 2, we performed a search of the medical literature for these interventions to identify those reporting clinically relevant outcomes. We prioritized systematic reviews of randomized clinical trials. In step 3, we classified the level of evidence and strength of recommendation based on the systematic reviews or RCT available using the GRADE system [17,18]. In addition to the quality of evidence and strength of recommendation, we selected checklist items that addressed clinically important, costly and/or common outcomes (e.g., death, severe sepsis), that were applicable to many ICU patients, that were often omitted at the individual level, and for which we could generate an objective question associated with a clear intervention. The frequency of compliance with the care process was estimated from literature data. We aimed to include no more than 10 to 12 items in the checklist [8]. Thus, we assessed each potential recommendation using the criteria listed below to decide which items to include in the pilot version of the checklist:

1. What is the relevance of the outcome(s) affected by the checklist item?
  ( ) Critical [e.g., death] ( ) Important ( ) Moderate [e.g., pressure ulcer]
2. Is the recommendation strong? Consider the determinants of the strength of recommendation:
  - Level of evidence (GRADE: risk of bias, inconsistency, inaccuracy, indirect evidence, publication bias)
    ( ) High ( ) Moderate ( ) Low ( ) Very Low
  - Is the balance between desirable and undesirable effects (adverse events and discomfort) favorable?
    ( ) Highly favorable
    ( ) Advantages in general higher than disadvantages
    ( ) Close balance of advantages and disadvantages
  - Costs (allocation of resources: training, human resources [complex interventions], financial resources)
    ( ) High ( ) Low
  - Variability (or uncertainty) in the values and preferences
    ( ) High ( ) Low
  Based on the above mentioned considerations, the strength of recommendation is:
  ( ) Strong ( ) Weak
3. Is it applicable to most ICU patients?
  ( ) All [100%] ( ) Many [30 to <100%] ( ) Few [<30%]
4. Are complications common, serious and costly?
  ( ) Meets three criteria ( ) Two criteria ( ) One or less
5. Is omission common? (At the individual level, e.g., oral chlorhexidine is a common omission in ICUs, but, in the ICUs using chlorhexidine, omission is rare at the individual level)
  ( ) Yes ( ) No
6. Can we generate an objective question (recommendation) associated with a clear intervention?
  ( ) Yes ( ) No
  *Conclusion*: Should the item be included in the checklist?
  ( ) Yes ( ) No

At this stage, we decided to include all items listed above, except the systematic use of oral chlorhexidine and achieving optimal nutritional requirements. Oral hygiene with chlorhexidine decreases ventilator-associated pneumonia (VAP) in patients after cardiac surgery but may have a neutral effect on VAP in other critically ill patients and controversial effects on mortality [19,20]. Additionally, the Steering Committee initially opted not to include a recommendation for achieving optimal nutritional requirements because moderate-quality evidence suggests a neutral effect on patient-centered outcomes [21]. At this stage, the Steering Committee generated a version of the checklist with 10 items.

Step 4 consisted of iterative tests and minor revisions of the checklist. The objectives were to evaluate the language (if the items were clear, objective, and brief) and the time required for applying the checklist. After obtaining a pre-final version, we tested the pilot version in two ICUs. The mean time to apply the checklist was 5 min. We also asked the multidisciplinary teams of the ICUs to answer a brief survey on their perception of the checklist. Thirteen health care professionals answered. All professionals agreed that the checklist was clear, objective, and helped to improve patient care. Eleven of the thirteen agreed that the checklist was easy to use, fast to apply, and increased interaction between the multidisciplinary team.

**Figure 2:**
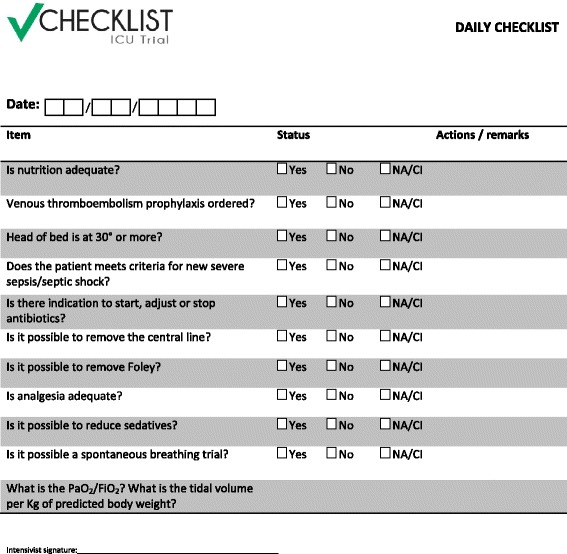
**Daily rounds checklist.**

In the final step, we presented the evaluation of each item under consideration for the checklist (Additional file 2) and submitted it to the investigators for approval during the experimental arm investigators meeting. All items suggested by the Steering Committee in the preliminary version of the checklist were approved by the investigators, except that most investigators demanded that a recommendation for achieving optimal nutritional requirements was included. The final version of the checklist is presented in Figure 2.

The checklists are arranged in a paper notebook (one per patient) with a daily list on each page, as most ICUs do not have electronic health record systems. During the multidisciplinary visit, the checklist items are read aloud by the nurse and answered by participants of the visit. The checklist is applied at least once on all week days preferably in the mornings, although we strongly suggest applying it also on weekend days.

#### Daily goals and clinician prompting

**Figure 3:**
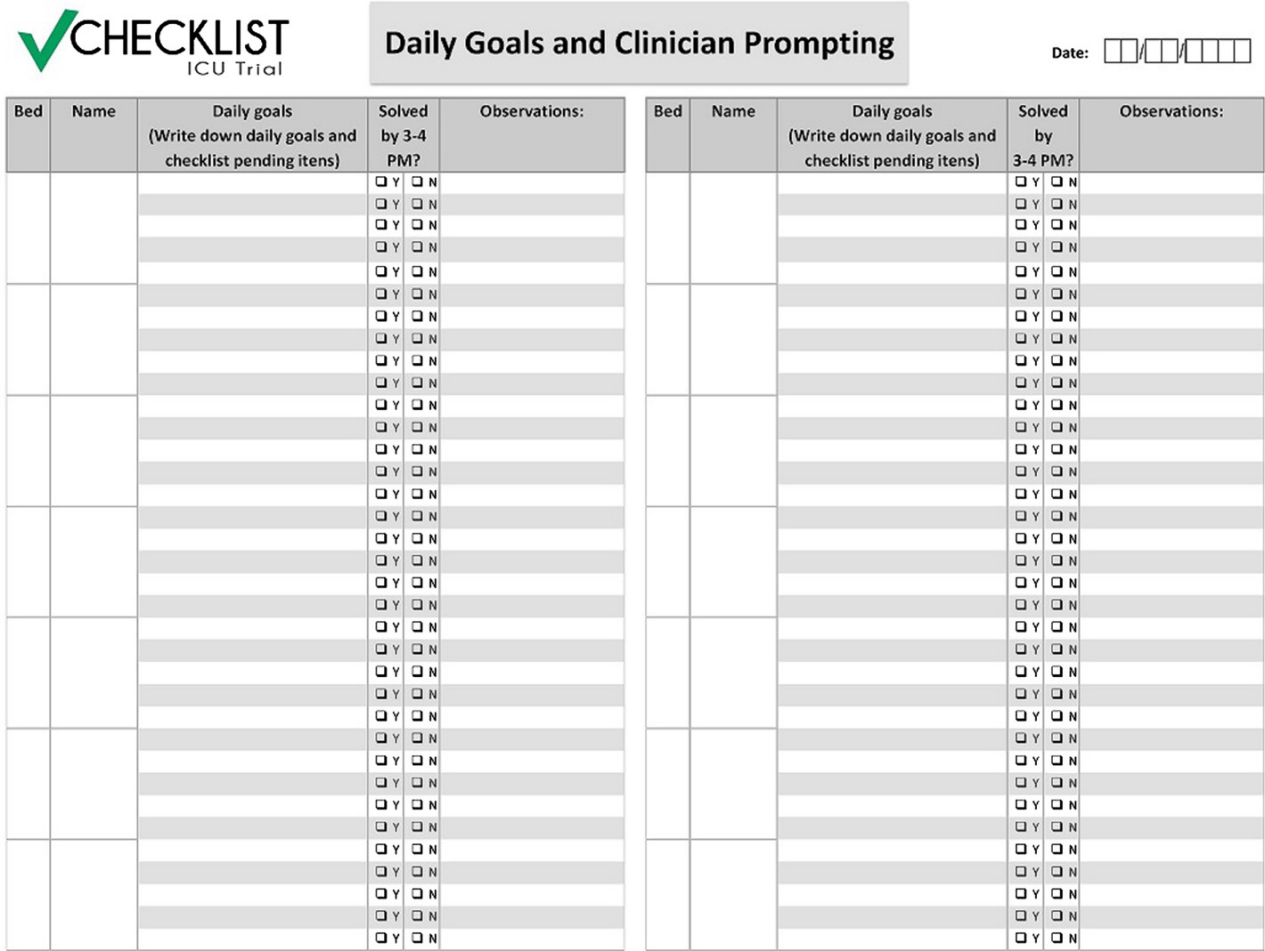
**Daily goals and clinical prompting form.**

During the clinical discussion of each patient and the application of the checklist, the intensivists write down the daily goals in a standardized form and read them aloud to the team (Figure 3). Every afternoon between 3 and 5 pm, a nurse reviews the daily goals and takes note of any pending items. Subsequently, the nurse prompts the on-call physician, requesting solutions for these pending items.

#### Strategies for implementing the study interventions

Based on successful deployment of checklists in prior settings [9,10,13], we were keen to encourage a specific deployment strategy that would help the checklist to be successful. Specifically, we wished to create a flat hierarchy, empowering the entire team to actively participate in rounds. That is, we expected to leverage the checklist potential not only to make sure important care interventions are not forgotten but also to promote a healthy team dynamics in which the team leader (usually the senior attending) listens well to the staff. Our implementation strategy to facilitate the use of the checklists and clinician prompting in the ICUs of the experimental arm are grouped in seven categories detailed in Table 1.

**Table 1:** **Strategies for implementing the interventions of the study**

**Categories**	**Strategies**
Experimental arm investigators meeting	The medical director and nursing director of all ICUs randomized to the experimental arm are invited to attend a one-day meeting, The objectives of the meeting are to present the rationale for the study interventions and the results of the observational phase (baseline results of ICU characteristics, adherence to healthcare processes, patient outcomes and safety climate), to vote on the items for the checklist and to provide training on the use of the study interventions.
Initiation visit of randomized phase	All sites in the experimental arm are visited by one intensivist from the Steering Committee. In these visits, we present the study design, adherence and clinical results of the observational phase, the checklist and definition of targets for improvement to the multidisciplinary ICU team. We also participate in multidisciplinary rounds to train the teams on the application of the checklist and definition of daily goals.
Audit & feedback	We generate monthly reports regarding the rates of adherence of selected processes of care using data collected on the study electronic case report forms. These reports include goals for each process of care so that we can classify the rate of adherence as “achieve the goal,” “close to the goal” or “do not achieve the goal”. Goals are defined based on compliance rates with the care processes obtained in the observational phase. We send these reports and schedule monthly conferences to discuss them with the ICU nursing and physician directors.
Contacts with ICU medical and nursing directors	The coordination center contacts the ICU medical and nursing directors if the checklist or clinician prompting is not being used regularly.
Study website	A study website is available with articles, study materials, videos and a forum to post questions, share experiences and images such as photos of the rounds.
Active reminders	We send SMS messages one to three times a week in the morning to staff from all experimental group ICUs to remind them of the time of the daily visit with the checklist and in the afternoon to remind them about clinician prompting.
Videos	Videos are presented in the training visits and are available on the study website, accessible only by health professionals working at the experimental arm ICUs. The videos contain material on how to use the checklist, how not to use the checklist and two video testimonials of well-known opinion leaders (Mr Paul O’Neill and Dr Derek Angus) that focus on successful quality improvement experiences, patient safety, leadership and team communication.

#### Control arm

The ICUs randomized to the control arm maintain usual care. That is, they are supposed to maintain multidisciplinary rounds, but we recommend that they not implement checklists during the trial.

### Data collection and management

All data, including outcome data, are collected by a health care professional, either a physician or a non-physician, who does not provide care for ICU patients and who is, preferentially, a staff member of the infection control department.

Data are entered by each center team in an electronic case report form via the Internet. Training and an instruction manual for using the system are provided to the investigators.

We apply the following procedures to ensure data quality: health care professionals who collect data attend a training session before the start of the study to standardize data collection; the investigators are able to contact the study coordinating center to solve issues or problems; data entry into our electronic case reports are subject to checks for missing data, possible or non-permitted value ranges, and logic checks. The system reports any problems at the time of data entry. All data entered into the system are reviewed by the data manager of the study, who sends requests for the correction of inconsistencies or for missing data to the investigators. Statistical techniques to identify inconsistencies are applied periodically (about every 2 weeks). The coordinating center also reviews detailed reports on screening, inclusion, follow-up, and data consistency and completeness every month.

### Outcomes

Our primary outcome is in-hospital mortality, truncated at 60 days. As we consider only patients with lengths of ICU stay longer than 48 h for the analysis, we assess only deaths occurring after that period. We chose this approach because deaths occurring within the first 48 h of ICU admission are unlikely to be affected by the interventions on the checklist.

Our secondary outcomes reflect the adherence to processes of care, patients’ clinical results, and safety culture. The secondary outcomes that demonstrate adherence to the appropriate care processes are as follows (Additional file 3): head of bed elevated at 30° in eligible patients; adequate prevention of venous thromboembolism; rate of central line catheter use; rate of indwelling urinary catheter use; patient-days under light sedation or alert and calm (RASS −3 to 0) in patients on mechanical ventilation; tidal volume ≤ 8 mL/kg in patients on mechanical ventilation; and rate of patients receiving enteral or parenteral feeding.

To assess the safety culture, we use the validated Brazilian-Portuguese version of the Safety Attitudes Questionnaire (SAQ) [22,23]. Adequate safety culture, as assessed by this questionnaire, is associated with indicators that demonstrate patient safety such as rates of hospital infection [24,25]. The questionnaire also has good psychometric properties (Cronbach’s alpha 0.7 to 0.8) and is sensitive to assess individual safety attitudes [24,25]. All health care professionals (physicians, nurses, practical nurses, respiratory therapists, nutritionists, psychologists, speech therapists, pharmacists, occupational therapists, social worker, etc.) of the participating ICUs are invited to complete the SAQ. In order to ensure most staff completes SAQ and also unbiased answers, we assure anonymity. Thus we will not record names or identification codes in the questionnaires. We ask the principal investigators to indicate the number of professionals at their ICUs. The goal of the ICUs is to collect questionnaires from at least 75% of staff members.

The secondary outcomes that reflect clinical results are as follows (Additional file 4): ICU mortality; mechanical ventilation-free days between day 1 and day 28; central line-associated bloodstream infection (CLABSI) rate; VAP rate; urinary tract infection (UTI) rate; length of ICU stay; and length of hospital stay.

### Adjudication of VAP and CLABSI

CLABSI and UTI are defined according to the 2008 Centers for Disease Control and Prevention (CDC) and National Healthcare Safety Network criteria [26]. VAP is defined according to the 2013 CDC criteria [27].

Daily data for the surveillance of ventilator-associated events of all patients on mechanical ventilation are sent to the coordinating center on a standardized form. Based on these data, a research nurse on the coordinating center identifies cases of VAP. Those cases are adjudicated by a blinded intensivist from the coordinating center.

Investigators send the results of all blood cultures from patients with venous central lines to the coordinating center, and in case of positive cultures, information regarding other criteria for CLABSI (whether there are other probable sites for the infection, and for skin contaminants, whether there are signs and symptoms and how many blood cultures are positive for the same microorganism) is also sent. CLABSI cases are also adjudicated in a process similar to that of the VAP cases.

### Randomization and allocation concealment

The unit of randomization is the ICU. In hospitals with more than one ICU, the ICUs are considered separate units of randomization provided that the care teams are completely different. If the health care teams are the same in the various ICUs of a single hospital, all ICUs are considered as a single cluster. The stratified and block randomization list is generated by a statistician of HCor Research Institute using an appropriate statistical package. Stratification is performed according to the median of in-hospital mortality determined in the observational preparatory phase. To ensure allocation concealment, the statistician who prepares the list receives only the identification code of the unit and is not aware of the identity of the ICU. The allocation list is then sent to the research manager, who informs the ICUs about their randomization status.

### Sample size

We plan to include at least 102 ICUs and 60 patients per ICU. With 102 ICUs and an average of 50 patients per unit, the study has a power of 90% and a type I error of 5% to detect an absolute reduction in the in-hospital mortality truncated at day 60 of 6% (from 30% in the control arm to 24% in the experimental arm), considering a coefficient of variation, K, of 0.25 [28].

### Statistical analysis

All analysis will be described in full detail in a statistical analysis plan, which will be submitted to publication before we lock the database and start analyses. The primary statistical analyses will be conducted according to the “intention-to-treat” principle. The primary outcome, in-hospital mortality truncated at 60 days, will be analyzed using random effects logistic regression [29], considering a fixed effect intercept for the strata [30] and adjusting for ICUs’ standardized mortality ratio (calculated with Simplified Acute Physiology Score 3 (SAPS3)) observed in the observational phase and patients’ SAPS3 score observed in the randomized phase. The purpose of the random effects regression models is to account for the correlation of observations of individuals within clusters. Likewise, in all other analyses to examine the effect of trial interventions on outcomes, we will use generalized linear mixed models including baseline values of the outcome variable at the ICU level determined in the observational phase as cofactors. We will use multiple mediation models to quantify the indirect effects of the use of checklists and clinician prompting on mortality mediated by the target care processes of the checklist and changes in the safety culture [31].

### Blinding

It is not feasible to blind the researchers, health care teams, or patients to the study intervention. However, the intensivists who adjudicate cases of VAP and CLABSI are blinded.

### Ethical aspects and good clinical practices

The study is carried out in accordance with Resolution no. 466/2012 and additional rulings by the Brazilian National Health Council/Ministry of Health, the Helsinki Declaration and all of its revisions and changes, and the Document of the Americas. The study protocol was approved by the coordinating site research ethics committee (approval no. CAAE 11673812.3.1001.0060) and the research ethics committees of all participant institutions. We required written institutional approval. The Institutional Approval Form was analyzed and approved by the research ethics committee and signed by the Director of each participating institution and the coordinator of the ICU. Obtaining the informed consent of patients raises logistical and methodological problems in health care quality improvement studies using cluster randomization [32]. Thus, we obtained a waiver for the requirement to obtain informed consent from patients or their relatives in all participating sites. We obtain written informed consent from ICU health care personnel before asking them to fill in the Safety Attitudes Questionnaire.

Data collection for the characterization of hospitals and ICUs is performed anonymously to prevent later identification of the units. Data is reported in an aggregate manner. Patient identification data are not submitted to the coordinating center of the study.

### Study organization

The teams of the Research Institute at Hospital do Coracao (IEP-HCor), D’Or Institute for Research and Education (IDOR) and Hospital Samaritano São Paulo are the sponsors and coordinators of the study, in association with the Brazilian Research in Intensive Care Network (BRICNet). BRICNet is an independent and collaborative Brazilian network focused on the performance of clinical studies in the field of intensive care medicine. The Latin American Sepsis Institute (LASI) also participates in the coordination of the trial. The study is supported by the Brazilian Association of Intensive Care Medicine (AMIB) via its research network (AMIB-Net).

The Steering Committee is responsible for the overall study supervision, assisting in developing the study protocol and preparing the final manuscript. The Steering Committee members are investigators, intensivists, and epidemiologists trained in designing and conducting multicenter randomized clinical trials.

Because of the relatively short duration of the intervention, we do not carry out interim analyses. Accordingly, there is no data monitoring committee.

### Trial status

As of November, 2014, we have enrolled more than 13,000 patients in 118 ICUs. Approximately half of the patients were studied during the observational pre-randomization phase between September 2013 and March 2014, and half were included in the randomized phase between April 2014 and September 2014. Patient follow-up will finish in November 2014. Also, 6,498 staff answered the SAQ in the observational phase and similar figures in the randomized phase (SAQ collection will be closed on November 2014). The study team remains blinded to results at this time, that is, we plan to close the database and to start analysis in December 2014.

## Discussion

The CHECKLIST-ICU Trial is a pragmatic cluster randomized trial that assesses whether a multifaceted quality improvement intervention that includes checklists, assessment of daily care goals during multidisciplinary rounds, and clinician prompting can improve the in-hospital mortality of critically ill patients. The trial also assesses whether the intervention can improve the ICU safety culture, processes of care, and other relevant clinical outcomes in critically ill patients. Finally, if the intervention effectively decreases in-hospital mortality, we will also provide insights on the relative contribution of the potential mediators: change in safety culture domains versus improvement in selected processes of care.

Medical culture is entrenched and highly hierarchical, which may inhibit collaborative multidisciplinary work [16]. A hierarchical ICU culture may be even more marked in nations outside Europe, North America, Australia, and New Zealand [33], and this may be one possible reason for the worse ICU outcomes in those countries. A key question we aim to clarify with this study is whether the checklist works not only through higher compliance with its items but also through promotion of improved teamwork and flattened hierarchy.

To our knowledge, this is the largest cluster randomized trial in critically ill patients, involving 118 intensive care units and more than 13,000 patients. Additionally, about 6,500 health care professionals answered the Safety Attitudes Questionnaire both in the observational and randomized phases. This will allow for precise assessment of the effect of the multifaceted intervention not only on processes of care but also on the safety culture and clinical outcomes.

Scales et al. conducted a cluster randomized trial involving 15 community intensive care units in Ontario [34]. The trial assessed a multifaceted intervention involving a videoconference-based forum with audits and feedback, expert-led educational sessions, and the dissemination of algorithms to improve adherence to six processes of care. There was moderate improvement in two practices, semi-recumbent positioning and precautions to prevent catheter-related bloodstream infections. This study is a benchmark for quality improvement research in ICUs; however, due to its sample size, it did not assess the effect on patient clinical outcomes.

Our study has several design features that merit attention. It has a cluster randomized design, which limits selection bias and the effects of secular trends. The strategies for implementing the multifaceted intervention of the study were carefully designed and thoroughly applied. Data collection is carried out by a professional not involved with the care of ICU patients, limiting biases in the data assessment and avoiding changes in processes of care that are not adequate when observed. Our study design, with assessment of safety culture with the SAQ and statistical analysis plan with multiple mediation models, will allow us to test the hypothesis that the checklist works not only through higher compliance with its items but also though promotion of improved teamwork and flattened hierarchy. Finally, the quality improvement strategies, if proven beneficial, can be incorporated and retained in the ICUs after the study ends. ICUs in the control arm will also start the study interventions soon after the end of randomized phase.

One limitation of our trial is the short time to evaluate the effect of the quality improvement intervention. It is possible that there is a progressive improvement in processes of care and clinical outcomes over the months, in particular, due to changes in the safety culture of the staff. The maximum time to include and follow up 60 patients after the intervention has started is 6 months, but ICUs with a large turnover of eligible patients can finish inclusion and follow-up in shorter time frames.

In conclusion, if this study finds that the implementation of the interventions, including the use of checklists during daily multidisciplinary rounds and clinician prompting, is able to reduce mortality and/or other relevant outcomes of patients, these interventions might be widely used in intensive care medicine, even in settings with limited resources.

## Additional files

Additional file 1:
**CONSORT checklist.** CONSORT 2010 checklist of information to include when reporting a cluster randomised trial. Additional file 2:
**CHECKLIST Elaboration.** Step 3—Assessment of quality of evidence, strength of recommendation and criteria to include an item in the checklist. Additional file 3:
**Outcomes.** Outcomes reflecting care processes. Additional file 4:
**Clinical outcomes.**

## Abbreviations

AMIB: Brazilian Association of Intensive Care Medicine
AMIB-Net: AMIB Rresearch Network
BRICNet: Brazilian Research in Intensive Care Network
CDC: Centers for Disease Control and Prevention
CLABSI: central line-associated bloodstream infection
ICU: intensive care units
IDOR: D’Or Institute for Research and Education
IEP-HCor: Research Institute at Hospital do Coracao
IQR: interquartile range
LASI: Latin American Sepsis Institute
PROADI-SUS: Program to Support Institutional Development of Universal Health System
SAPS3: Simplified Acute Physiology Score 3
SAQ: Safety Attitudes Questionnaire
SOFA: Sequential Organ Failure Assessment
SD: standard deviation
UTI: urinary tract infection
VAP: ventilator-associated pneumonia
