# Erratum to: A cluster randomized trial of a multifaceted quality improvement intervention in Brazilian intensive care units: study protocol

**DOI:** 10.1186/s13012-015-0288-z

**Published:** 2015-08-13

**Authors:** The CHECKLIST-ICU Investigators and the BRICNet

**Affiliations:** Research Institute - Hospital do Coração (IEP– HCor), Rua Abílio Soares 250, 12th floor, SP, CEP: 04005-000, São Paulo, Brazil

## Erratum

Due to an error during the production process Alexandre Cavalcanti was incorrectly added to the author list of the original article [1]. The author list has now been updated in the original published article from:

Alexandre Cavalcanti and The CHECKLIST-ICU Investigators and the BRICNet

To:

The CHECKLIST-ICU Investigators and the BRICNet

BioMed Central apologises to the authors and readers for this error and any inconvenience this has caused.
