# Supplementary material for: A cluster randomized trial of a multifaceted quality improvement intervention in Brazilian intensive care units: study protocol
Source: Implement Sci. 2015 Jan 13;10:8. doi: 10.1186/s13012-014-0190-0 (PMC4342101; doi:10.1186/s13012-014-0190-0)
Supplement: Additional file 1: — CONSORT checklist. CONSORT 2010 checklist of information to include when reporting a cluster randomised trial. [file 13012_2014_190_MOESM1_ESM.docx]

**CHECKLIST Elaboration - Step 3 – Assessment of quality of evidence, strength of recommendation and criteria to include an item in the checklist**

**CONTENTS**

| Venous thromboembolism prophylaxis | _______________________________ | 2 |
| --- | --- | --- |
| Screening for severe sepsis | _______________________________ | 6 |
| Need to start, adjust or discontinue antibiotics | _______________________________ | 9 |
| Venous central line removal | _______________________________ | 14 |
| Urinary catheter removal | _______________________________ | 18 |
| Low tidal volume for ARDS patients | _______________________________ | 22 |
| Low tidal volume for patients without ARDS | _______________________________ | 27 |
| Semirecumbent position | _______________________________ | 32 |
| Pain control in the ICU | _______________________________ | 38 |
| Light sedation | _______________________________ | 42 |
| Discontinuation of mechanical ventilation | _______________________________ | 45 |
| Oral hygiene with chlorhexidine | _______________________________ | 48 |
| Achieving optimal nutritional requirements | _______________________________ | 52 |

Venous thromboembolism prophylaxis

**Clinical Question:**

Should any heparin (LDUH, LMWH) vs placebo be used for preventing venous thromboembolism in critically ill adult patients?

# Identify and critically appraise systematic reviews of randomized trials on the topic

The Antithrombotic Therapy and Prevention of Thrombosis, 9th ed: American College of Chest Physicians Evidence-Based Clinical Practice Guidelines issued recommendations for TEV prevention in critically ill patients [1]. These guidelines included systematic reviews for the topics considered.

Only five five RCTs have assessed pharmacologic prophylaxis in critically ill patients. One compared low dose unfractioned heparin (LDUH) to placebo, one low molecular weight heparin (LMWH) to placebo, and three LDUH to LMWH. Only one trial provided evidence for the comparison between any heparin vs placebo, and the estimates of effect were very imprecise due to low number of events. The relative risk for symptomatic deep venous thrombosis was 0.86 (95%CI 0.59-1.25) and for pulmonary embolism was 0.73 (95%CI 0.26-2.11). Thus the quality of evidence supporting an effect of prophylaxis with any heparin on DVT was considered moderate due to serious imprecision. The quality of evidence for preventing pulmonary embolism was considered very low due to very serious imprecision and indirectness (outcomes were a mix of symptomatic and asymptomatic events).

Regarding the comparison between LMWH versus LDUH, there was a reduction in pulmonary embolism. However the magnitude of effect was small and effect estimates were imprecise. Furthermore, most events were observed in a trial which performed screening compression ultrasonography on all enrolled patients, which differs from real other practice[2]. If the asymptomatic DVTs were left undiagnosed and untreated, with some progressing to DVT, the treatment effect would likely be different. Thus the ACCP Guidelines did not issue a recommendation favoring LMWH.

For critically ill patients who are bleeding, or are at high risk of major bleeding, the ACCP Guidelines suggested mechanical thromboprophylaxis with gradual compressing stocks or intermittent pneumatic compression[1]. This weak recommendation is based on evidence extrapolated from trials assessing mechanical methods in surgical patients[3].

# Strength of recommendation

**Table 1.** Considerations to determine the strength of recommendation

| Factor | Comment |
| --- | --- |
| Balance between desirable and undesirable effects | Prophylaxis of venous thromboembolism with LMWH or LDUH may reduce the risk of deep venous thrombosis. However, there may be an increased risk of bleeding.  Mechanical methods may be used in patients at high risk for bleeding, but effectiveness is less certain. |
| Quality of evidence | Moderate evidence suggests heparins are effective for preventing deep venous thrombosis in critically ill patients. Evidence of effect on pulmonary embolism is very low. |
| Values and preferences | We assume that there is considerable variability in patients and relatives preferences between a likely benefit for preventing DVT, and, with less certainty pulmonary embolism, but at a increased risk of bleeding. |
| Costs (resource allocation) | Low. |

The 9^th^ edition of the ACCP Guidelines of Antithrombotic Therapy and Prevention ofThrombosis issued a weak recommendation in favor of prophylaxis with LMWH or LDUH in critically ill patients. Also, the recommendation for mechanical prophylaxis for VTE was weak and limited to patients unable to receive heparins due to active bleeding or high risk of bleeding.

**Table 1.** Criteria to include venous thromboembolism prophylaxis in the daily round checklist

| 1. What is the relevance of the outcome(s) affected by the checklist item?  - Reduction in pulmonary embolism (PE)   ( ) Critical [e.g.: death] ( x ) Important ( ) Moderate [e.g.: pressure ulcer]   - Reduction in deep venous thrombosis   ( ) Critical [e.g.: death] ( ) Important ( x ) Moderate [e.g.: pressure ulcer] |
| --- |
| 1. Is the recommendation strong? Consider the determinants of the strength of recommendation:    1. Level of evidence (GRADE: risk of bias, inconsistency, inaccuracy, indirect evidence, publication bias)   Reduction in PE: ( ) High ( ) Moderate ( ) Low ( x ) Very Low  Reduction in DVT: ( ) High ( x ) Moderate ( ) Low ( ) Very Low   - 1. Is the balance between desirable and undesirable effects (adverse events and discomfort) favorable?   ( ) Highly favorable  ( x) Advantages in general higher than disadvantages  ( ) Close balance of advantages and disadvantages   - 1. Costs (allocation of resources: training, human resources [complex interventions], financial resources)   ( ) High ( x ) Low   - 1. Variability (or uncertainty) in the values ​​and preferences   ( ) High ( x ) Low  Based on the above mentioned considerations, the strength of recommendation is:  (x ) Strong ( ) Weak |
| 1. Is it applicable to most ICU patients?   ( ) All [100%] ( x ) Many [30 to <100%] ( x ) Few [<30%] |
| 1. Are complications common, serious and costly?   ( ) Meets three criteria ( x ) Two criteria ( ) One or less |
| 1. Is omission common? (at the individual level, e.g.: oral chlorhexidine is a common omission in ICUs, but, in the ICUs using chlorhexidine, omission is rare at the individual)   ( x ) Yes ( ) No |
| 1. Can we generate an objective question (recommendation) associated with a clear intervention?   ( x ) Yes ( ) No |
| **Conclusion:** Should the item be included in the checklist?  ( x ) Yes ( ) No |

Reference List

1. Kahn SR, Lim W, Dunn AS, Cushman M, Dentali F, Akl EA, Cook DJ, Balekian AA, Klein RC, Le H et al.: **Prevention of VTE in nonsurgical patients: Antithrombotic Therapy and Prevention of Thrombosis, 9th ed: American College of Chest Physicians Evidence-Based Clinical Practice Guidelines.** *Chest* 2012, **141:**e195S-e226S.

2. Cook D, Meade M, Guyatt G, Walter S, Heels-Ansdell D, Warkentin TE, Zytaruk N, Crowther M, Geerts W, Cooper DJ et al.: **Dalteparin versus unfractionated heparin in critically ill patients.** *N Engl J Med* 2011, **364:**1305-1314.

3. Kakkos SK, Caprini JA, Geroulakos G, Nicolaides AN, Stansby GP, Reddy DJ: **Combined intermittent pneumatic leg compression and pharmacological prophylaxis for prevention of venous thromboembolism in high-risk patients.** *Cochrane Database Syst Rev* 2008,CD005258.

Screening for severe sepsis

**Clinical Question:**

The use of screening tools for severe sepsis reduces mortality?

# Identify and critically appraise systematic reviews of randomized trials on the topic

We have found no systematic review on the topic.

The early identification of sepsis using a sepsis screening tool with initiation of appropriate evidence-based therapies has been shown to decrease sepsis-related mortality in several “before-after” studies [1-6]. Although, there is no evidence from randomized trials, several studies suggest that reducing the time between organ dysfunction and the initiation of antibiotics and other element of 3 and 6 hour bundle is critical to reduce mortality [7-10]. We considered the quality of evidence as low, as it is based on several before-after studies.

# Strength of recommendation

**Table 1.** Considerations to determine the strength of recommendation

| Factor | Comment |
| --- | --- |
| Balance between desirable and undesirable effects | Screening for severe sepsis probably reduces the time between organ dysfunction and the initiation of sepsis treatment, thus it likely improves clinical outcomes of sepsis patients. Conversely, there are no substantial inconveniences. |
| Quality of evidence | Low quality |
| Values and preferences | Clear preference for early diagnosis of severe sepsis and the possibility to improve clinical outcomes. |
| Costs (resource allocation) | Low. |

**Table 1.** Criteria to include “screening for sepsis” in the daily round checklist

| 1. What is the relevance of the outcome(s) affected by the checklist item?  - mortality   ( x ) Critical [e.g.: death] ( ) Important ( ) Moderate [e.g.: pressure ulcer] |
| --- |
| 1. Is the recommendation strong? Consider the determinants of the strength of recommendation:    1. Level of evidence (GRADE: risk of bias, inconsistency, inaccuracy, indirect evidence, publication bias)   ( ) High ( ) Moderate ( x ) Low ( ) Very Low   - 1. Is the balance between desirable and undesirable effects (adverse events and discomfort) favorable?   ( x ) Highly favorable  ( ) Advantages in general higher than disadvantages  ( ) Close balance of advantages and disadvantages   - 1. Costs (allocation of resources: training, human resources [complex interventions], financial resources)   ( ) High ( x ) Low   - 1. Variability (or uncertainty) in the values ​​and preferences   ( ) High ( x ) Low  Based on the above mentioned considerations, the strength of recommendation is:  (x ) Strong ( ) Weak |
| 1. Is it applicable to most ICU patients?   ( ) All [100%] ( x ) Many [30 to <100%] ( x ) Few [<30%] |
| 1. Are complications common, serious and costly?   ( x ) Meets three criteria ( ) Two criteria ( ) One or less |
| 1. Is omission common? (at the individual level, e.g.: oral chlorhexidine is a common omission in ICUs, but, in the ICUs using chlorhexidine, omission is rare at the individual)   ( x ) Yes ( ) No |
| 1. Can we generate an objective question (recommendation) associated with a clear intervention?   ( x ) Yes ( ) No |
| **Conclusion:** Should the item be included in the checklist?  ( x ) Yes ( ) No |

Reference List

1. Levy MM, Dellinger RP, Townsend SR et al. The Surviving Sepsis Campaign: results of an international guideline-based performance improvement program targeting severe sepsis. Crit Care Med 2010;38(2):367-374.

2. Levy MM, Dellinger RP, Townsend SR et al. The Surviving Sepsis Campaign: results of an international guideline-based performance improvement program targeting severe sepsis. Crit Care Med 2010;38(2):367-374.

3. Micek ST, Roubinian N, Heuring T et al. Before-after study of a standardized hospital order set for the management of septic shock. Crit Care Med 2006;34(11):2707-2713.

4. Nguyen HB, Corbett SW, Steele R et al. Implementation of a bundle of quality indicators for the early management of severe sepsis and septic shock is associated with decreased mortality. Crit Care Med 2007;35(4):1105-1112.

5. Thiel SW, Asghar MF, Micek ST, Reichley RM, Doherty JA, Kollef MH. Hospital-wide impact of a standardized order set for the management of bacteremic severe sepsis. Crit Care Med 2009;37(3):819-824.

6. Castellanos-Ortega A, Suberviola B, Garcia-Astudillo LA et al. Impact of the Surviving Sepsis Campaign protocols on hospital length of stay and mortality in septic shock patients: results of a three-year follow-up quasi-experimental study. Crit Care Med 2010;38(4):1036-1043.

7. Kumar A, Roberts D, Wood KE et al. Duration of hypotension before initiation of effective antimicrobial therapy is the critical determinant of survival in human septic shock. Crit Care Med 2006;34(6):1589-1596.

8. Bloos F, Thomas-Ruddel D, Ruddel H et al. Impact of compliance with infection management guidelines on outcome in patients with severe sepsis: a prospective observational multi-center study. Crit Care 2014;18(2):R42.

9. Azuhata T, Kinoshita K, Kawano D et al. Time from admission to initiation of surgery for source control is a critical determinant of survival in patients with gastrointestinal perforation with associated septic shock. Crit Care 2014;18(3):R87.

10. Beck V, Chateau D, Bryson GL et al. Timing of vasopressor initiation and mortality in septic shock: a cohort study. Crit Care 2014;18(3):R97.

Need to start, adjust or discontinue antibiotics

**Question:** Considering whether it is needed starting, adjusting or discontinuing antibiotics can improve survival, decrease toxicity, costs and resistance?

# Identify and critically appraise systematic reviews of randomized trials on the topic

Timely initiation of antibiotics in patients with severe sepsis or septic shock has been shown to improve survival in some observational studies [7,11]. The GRADE quality of evidence is low, as it is based on observational studies only.

A systematic review found no randomized trial assessing de-escalation of antibiotics in patients with severe sepsis or septic shock [12]. Another systematic review not limited to patients with severe sepsis included mostly observational studies, only 3 were randomized trials. It found a decrease in costs, antibiotics adverse events and resistance rates. There was no effect on nosocomial infection rates, length of stay or mortality [13]. Therefore, the quality of evidence is low

Some systematic reviews assessed the safety of shorter antibiotics courses for ventilator-associated pneumonia, pyelonephritis or bacteremia[14-16]. All suggested equivalence of the shorter course. However, in general, effect estimates on clinical outcomes were imprecise. Thus, the quality of evidence is deemed to be moderate.

A systematic review of randomized trials of procalcitonin to discontinue antibiotics in patients with respiratory infections found neutral effect on mortality (odds ratio 0.91; 95% CI 0.7 to 1.19). Quality of evidence was considered moderate due to some concern regarding risk of bias and imprecision. There was a decrease in treatment failure (odds ratio 0.83; 95% CI 0.71 to 0.97) and a decrease in duration of antibiotics (mean difference 3.47; 95% CI 3.78 to 3.17), both with moderate quality of evidence (due to risk of bias in primary studies).

# Strength of recommendation

**Table 1.** Considerations to determine the strength of recommendation

| Factor | Comment |
| --- | --- |
| Balance between desirable and undesirable effects | Timely initiation of adequate antibiotics may improve survival in severe sepsis and septic shock.  De-escalation of antibiotics may reduce antibiotics resistance and costs. No evidence of effect on mortality, nosocomial infection rates or length of in-hospital stay.  Shorter courses of antibiotics are likely equivalent to longer courses in terms of mortality. |
| Quality of evidence | Low quality for timely initiation of adequate antibiotics on mortaliy (observational studies)  Low quality for de-escalation of antibiotics on resistance (systematic review of mixed randomized and non-randomized trials)  Moderate quality for shorter courses of antibiotics on mortality |
| Values and preferences | We assumed there is little variation for timely initiation as there is a very favorable balance between desirable and undesirable effects.  However, for de-escalation and shorter courses variation in preferences and values are higher. For the society perspective, removing pressure towards antibiotics resistance is a priority. However, for the patients/relatives and doctors perspectives, the risk of insufficient treatment may be a more important issue. |
| Costs (resource allocation) | Costs of timely initiation of adequate empiric antibiotics may be elevated depending on antibiotics choice. De-escalation and short courses of antibiotics may reduces costs. |

We consider timely initiation of appropriate antibiotics a strong recommendation and de-escalation or short courses of antibiotics as weak recommendations.

**Table 1.** Criteria to include “Need to start, adjust or discontinue anbiotics” in the daily round checklist

| 1. What is the relevance of the outcome(s) affected by the checklist item?  - Reduced mortality with timely initiation of adequate antibiotics. Equivalent mortality with shorter vs longer antibiotics course and with de-escalation   ( x ) Critical [e.g.: death] ( ) Important ( ) Moderate [e.g.: pressure ulcer]   - Reduction on antibiotics resistance for de-escalation   ( ) Critical [e.g.: death] ( x ) Important ( ) Moderate [e.g.: pressure ulcer] |
| --- |
| 1. Is the recommendation strong? Consider the determinants of the strength of recommendation:    1. Level of evidence (GRADE: risk of bias, inconsistency, inaccuracy, indirect evidence, publication bias)   Timely initiation of adequate antibiotics effect on mortality:  ( ) High ( ) Moderate ( x ) Low ( ) Very Low  De-escalation effects on mortality and resistance:  ( ) High ( ) Moderate ( x ) Low ( ) Very Low  Shorter courses of antibiotics for VAP, pyelonephritis or bacteremia and for procalcitoning guided antibiotics discontinuation  ( ) High ( x ) Moderate ( ) Low ( ) Very Low   - 1. Is the balance between desirable and undesirable effects (adverse events and discomfort) favorable?   ( ) Highly favorable  ( x ) Advantages in general higher than disadvantages  ( ) Close balance of advantages and disadvantages   - 1. Costs (allocation of resources: training, human resources [complex interventions], financial resources)   ( ) High ( x ) Low   - 1. Variability (or uncertainty) in the values ​​and preferences   Timely initiation of antibiotics: ( ) High ( x ) Low  De-escalation and shorter courses of antibiotics: ( x) High ( ) Low  Based on the above mentioned considerations, the strength of recommendation is:  Timely initiation of adequate antibiotics: (x ) Strong ( ) Weak  De-escalation and shorter courses of antibiotics: (x ) Strong ( ) Weak |
| 1. Is it applicable to most ICU patients?   ( ) All [100%] ( x ) Many [30 to <100%] ( x ) Few [<30%] |
| 1. Are complications common, serious and costly?   ( x ) Meets three criteria ( ) Two criteria ( ) One or less |
| 1. Is omission common? (at the individual level, e.g.: oral chlorhexidine is a common omission in ICUs, but, in the ICUs using chlorhexidine, omission is rare at the individual)   ( x ) Yes ( ) No |
| 1. Can we generate an objective question (recommendation) associated with a clear intervention?   ( x ) Yes ( ) No |
| **Conclusion:** Should the item be included in the checklist?  ( x ) Yes ( ) No |

Reference List

1. Gaieski DF, Mikkelsen ME, Band RA, Pines JM, Massone R, Furia FF, Shofer FS, Goyal M: **Impact of time to antibiotics on survival in patients with severe sepsis or septic shock in whom early goal-directed therapy was initiated in the emergency department.** *Crit Care Med* 2010, **38:**1045-1053.

2. Kumar A, Roberts D, Wood KE, Light B, Parrillo JE, Sharma S, Suppes R, Feinstein D, Zanotti S, Taiberg L et al.: **Duration of hypotension before initiation of effective antimicrobial therapy is the critical determinant of survival in human septic shock.** *Crit Care Med* 2006, **34:**1589-1596.

3. Silva BN, Andriolo RB, Atallah AN, Salomao R: **De-escalation of antimicrobial treatment for adults with sepsis, severe sepsis or septic shock.** *Cochrane Database Syst Rev* 2013, **3:**CD007934.

4. Kaki R, Elligsen M, Walker S, Simor A, Palmay L, Daneman N: **Impact of antimicrobial stewardship in critical care: a systematic review.** *J Antimicrob Chemother* 2011, **66:**1223-1230.

5. Havey TC, Fowler RA, Daneman N: **Duration of antibiotic therapy for bacteremia: a systematic review and meta-analysis.** *Crit Care* 2011, **15:**R267.

6. Pugh R, Grant C, Cooke RP, Dempsey G: **Short-course versus prolonged-course antibiotic therapy for hospital-acquired pneumonia in critically ill adults.** *Cochrane Database Syst Rev* 2011,CD007577.

7. Eliakim-Raz N, Yahav D, Paul M, Leibovici L: **Duration of antibiotic treatment for acute pyelonephritis and septic urinary tract infection-- 7 days or less versus longer treatment: systematic review and meta-analysis of randomized controlled trials.** *J Antimicrob Chemother* 2013, **68:**2183-2191.

Venous central line removal

**Clinical question:** Does daily assessment of venous central line removal decreases central line-associated bloodstream infection?

# Identify and critically appraise systematic reviews of randomized trials on the topic

Insertion of central venous catheters is among the most commonly performed procedures in ICU. Although venous central lines are indispensable in many cases, their use is associated with central line-associated bloodstream infections (CLABSI), increased costs and deaths.

Several before-after studies have applied bundles to decrease CLABSI with substantial success [1-4]. A key element of these bundles is daily consideration of central line removal. Other elements are hand washing before line placement; using full barrier precautions (full-body drape, hat, gloves, mask, and gown); avoiding line placement at the femoral site; and using chlorhexidine to cleanse the site [1]. An observer (usually a nurse) used a checklist to ensure that the clinician inserting the line followed the evidence-based practices and was empowered to stop the clinician to insert the line if violations were observed[1].

We have identified two systematic reviews assessing the effect of quality improvement interventions to reduce CLABSI[5,6].

The systematic review by Blot et al.[5] included 41 studies applying several quality improvement interventions to decrease CLABSI, mostly before-after studies, none was randomized. There was a decrease in CLABSI (odds ratio 0.39; 95%CI 0.33 to 0.46). Only 11 studies included the item “daily review of central line necessity”, although a subgroup analysis of these trials was not provided.

A Cochrane review by Flodgren et al. found 6 interrupted-time series assessing interventions to decrease CLABSI[6]. Due to marked heterogeneity the authors did not attempt meta-analysis. The effects on rate of infection were mixed and the effect sizes were generally small.

A cluster randomized controlled trial published in 2012 was not included in the previous review[7]. In this study, 45 ICUs were randomized to a experimental group, which received the Keystone collaborative bundle to prevent CLABSI[1], or to a control group which received no intervention. This study found an expressive reduction in the rate of CLABSI (rate ratio 0.19; 95% CI 0.06–0.57) providing strong support for the intervention.

We considered the quality of evidence in support of bundles to decrease CLABSI as high, based on the evidence generated by the cluster randomized trial plus the systematic review of before-after studies and interrupted-time series[5-7]. However, we have found no studies addressing specifically whether daily consideration for central line removal is effective to reduce CLABSI. Thus, we downgraded the evidence in support to daily assessment for central line removal as moderate.

# Strength of recommendation

**Table 1.** Considerations to determine the strength of recommendation

| Factor | Comment |
| --- | --- |
| Balance between desirable and undesirable effects | Daily assessment for central line removal is part of bundles of very effective bundles to prevent CLABSI. Inconveniences are the need for punctures to get peripheral line or the need to re-insert a central-line if the patient needs it once again.  The balance is favorable to removing unnecessary lines. |
| Quality of evidence | Moderate evidence for daily assessment of central line removal for preventing CLABSI. |
| Values and preferences | We assume that patients, relatives and ICU staff clearly prefer avoiding CLABSI than avoiding a peripheral puncture or the low risk of having the need to re-insert a central line. |
| Costs (resource allocation) | Low. |

We consider daily assessment for removal of unnecessary central lines a strong recommendation.

| 1. What is the relevance of the outcome(s) affected by the checklist item?  - Reduction of central line-associated bloodstream infection   ( ) Critical [e.g.: death] ( x) Important ( ) Moderate [e.g.: pressure ulcer] |
| --- |
| 1. Is the recommendation strong? Consider the determinants of the strength of recommendation:    1. Level of evidence (GRADE: risk of bias, inconsistency, inaccuracy, indirect evidence, publication bias)   ( ) High ( x ) Moderate ( ) Low ( ) Very Low   - 1. Is the balance between desirable and undesirable effects (adverse events and discomfort) favorable?   ( ) Highly favorable  ( x ) Advantages in general higher than disadvantages  ( ) Close balance of advantages and disadvantages   - 1. Costs (allocation of resources: training, human resources [complex interventions], financial resources)   ( ) High ( x ) Low   - 1. Variability (or uncertainty) in the values ​​and preferences   ( ) High ( x ) Low  Based on the above mentioned considerations, the strength of recommendation is:  ( x ) Strong ( ) Weak |
| 1. Is it applicable to most ICU patients?   ( ) All [100%] ( x ) Many [30 to <100%] ( ) Few [<30%] |
| 1. Are complications common, serious and costly?   ( ) Meets three criteria ( x ) Two criteria ( ) One or less |
| 1. Is omission common? (at the individual level, e.g.: oral chlorhexidine is a common omission in ICUs, but, in the ICUs using chlorhexidine, omission is rare at the individual)   ( x ) Yes ( ) No |
| 1. Can we generate an objective question (recommendation) associated with a clear intervention?   ( x ) Yes ( ) No |
| **Conclusion:** Should the item be included in the checklist?  ( x ) Yes ( ) No |

**Table 1.** Criteria to include venous central line removal assessment in the daily round checklist

Reference List

1. Pronovost P, Needham D, Berenholtz S, Sinopoli D, Chu H, Cosgrove S, Sexton B, Hyzy R, Welsh R, Roth G et al.: **An intervention to decrease catheter-related bloodstream infections in the ICU.** *N Engl J Med* 2006, **355:**2725-2732.

2. Bonello RS, Fletcher CE, Becker WK, Clutter KL, Arjes SL, Cook JJ, Petzel RA: **An intensive care unit quality improvement collaborative in nine Department of Veterans Affairs hospitals: reducing ventilator-associated pneumonia and catheter-related bloodstream infection rates.** *Jt Comm J Qual Patient Saf* 2008, **34:**639-645.

3. Coopersmith CM, Rebmann TL, Zack JE, Ward MR, Corcoran RM, Schallom ME, Sona CS, Buchman TG, Boyle WA, Polish LB et al.: **Effect of an education program on decreasing catheter-related bloodstream infections in the surgical intensive care unit.** *Crit Care Med* 2002, **30:**59-64.

4. Miller RS, Norris PR, Jenkins JM, Talbot TR, III, Starmer JM, Hutchison SA, Carr DS, Kleymeer CJ, Morris JA, Jr.: **Systems initiatives reduce healthcare-associated infections: a study of 22,928 device days in a single trauma unit.** *J Trauma* 2010, **68:**23-31.

5. Blot K, Bergs J, Vogelaers D, Blot S, Vandijck D: **Prevention of Central Line-Associated Bloodstream Infections Through Quality Improvement Interventions: A Systematic Review and Meta-analysis.** *Clin Infect Dis* 2014.

6. Flodgren G, Conterno LO, Mayhew A, Omar O, Pereira CR, Shepperd S: **Interventions to improve professional adherence to guidelines for prevention of device-related infections.** *Cochrane Database Syst Rev* 2013, **3:**CD006559.

7. Marsteller JA, Sexton JB, Hsu YJ, Hsiao CJ, Holzmueller CG, Pronovost PJ, Thompson DA: **A multicenter, phased, cluster-randomized controlled trial to reduce central line-associated bloodstream infections in intensive care units*.** *Crit Care Med* 2012, **40:**2933-2939.

Urinary catheter removal

**Clinical question:** Does daily check and removal of unnecessary urinary catheter improve clinical outcome of ICU patients?

# Identify and critically appraise systematic reviews of randomized trials on the topic

Urinary track infection (UTI) is the most common nosocomial infection corresponding to 30-40% of all hospital-acquired infections in United States [1] and can be almost exclusively attributed to instrumentation of urinary track, indeed more than 80% of UTIs are associated with an indwelling catheter. Catheter-associated urinary tract infection (CAUTI) has been associated with increased morbidity, mortality, hospital cost, and length of stay.

The frequency of patients with urinary catheters is high among hospitalized patients; 16% to 25% of hospitalized patients are in use of urinary catheters, but the prevalence rates in the critical care unit are still substantially higher at 67% to 76%[2].

The presence of a urinary catheter has been associated with a daily risk of developing UTI ranging from 3-7%. [3]. After 30 days of using a closed collecting system 100% of patients have bacteriuria [4]. In many cases, catheters are placed without appropriated indication, and health care professionals are not attentive to early removal leading to prolonged and unnecessary use.

It is well established that the risk for CAUTI is proportional to the permanence of the catheter. Therefore, reduction of catheter days is a central strategy of any CAUTI prevention program. However, how implement this strategy in critical care is a major challenge.

We found four recent systematic reviews addressing questions on preventive strategies to reduce CAUTI. One publication compared the effectiveness of different types of indwelling urethral catheters in reducing the risk of UTI [5], other analyzed the interventions to minimize the initial use of indwelling urinary catheters [6]. We considered these two systematic reviews out of the scope of this recommendation. The other two reviews were done by the same group of authors and the most recent review was an update from the previous one. We considered to this propose just the most recent [7].

The author identified 30 studies employing reminders and/or stop orders to removal of unnecessary urinary catheters[7]. These studies reported at least one CAUTI or urinary catheter use measures. The large majority (28) of studies used before-after designs, only one study was a randomised control trial (RCT) and one study was a non-randomized crossover trial.

The meta-analysis using 11 studies with the risk ratios for development of catheter-associated urinary tract infection, stratified by type of intervention (reminders and/or stop orders), indicated the rate of CAUTI (episodes per 1000 catheter-days) was reduced by 53% (rate ratio 0.47; 95% CI 0.30 to 0.64, p<0.001) with use of a reminder or stop order. No significant harm was noted.

The only RCT included in this meta-analysis was focused on catheterized general medicine patients. The urinary catheter use decreased in the intervention group (stop-order) compared patients receiving usual care by −1.34 days (95% CI −0.64 to−2.05 days, p<0.001). However, symptomatic CAUTI rates did not change (p=0.99).

We consider that the quality of evidence supporting the effect of reminders or stop orders on decreasing catheter use is high based on the meta-analysis of observational and the randomized trial. However, the quality of evidence supporting the effect of reminders/stop orders on CAUTI is moderate, as there is a large effect, although it is based on non-randomized trials.

# Strength of recommendation

**Table 1.** Considerations to determine the strength of recommendation

| Factor | Comment |
| --- | --- |
| Balance between desirable and undesirable effects | Daily assessment for urinary catheter removal may reduce urinary tract infection.  No significant harms are noted.  The balance is favorable to removing unnecessary lines. |
| Quality of evidence | Moderate evidence supports daily assessment for urinary catheter removal to prevent CAUTI. |
| Values and preferences | Strongly in favor of avoiding CAUTI. |
| Costs (resource allocation) | Low. |

We consider daily assessment for removal of unnecessary urinary catheters a strong recommendation.

**Table 1.** Criteria to include venous central line removal assessment in the daily round checklist

| 1. What is the relevance of the outcome(s) affected by the checklist item?  - Reduction of catheter-associated urinary tract infection   ( ) Critical [e.g.: death] ( ) Important ( x ) Moderate [e.g.: pressure ulcer] |
| --- |
| 1. Is the recommendation strong? Consider the determinants of the strength of recommendation:    1. Level of evidence (GRADE: risk of bias, inconsistency, inaccuracy, indirect evidence, publication bias)   ( ) High ( x ) Moderate ( ) Low ( ) Very Low   - 1. Is the balance between desirable and undesirable effects (adverse events and discomfort) favorable?   ( ) Highly favorable  ( x ) Advantages in general higher than disadvantages  ( ) Close balance of advantages and disadvantages   - 1. Costs (allocation of resources: training, human resources [complex interventions], financial resources)   ( ) High ( x ) Low   - 1. Variability (or uncertainty) in the values ​​and preferences   ( ) High ( x ) Low  Based on the above mentioned considerations, the strength of recommendation is:  ( x ) Strong ( ) Weak |
| 1. Is it applicable to most ICU patients?   ( ) All [100%] ( x ) Many [30 to <100%] ( ) Few [<30%] |
| 1. Are complications common, serious and costly?   ( ) Meets three criteria ( x ) Two criteria ( ) One or less |
| 1. Is omission common? (at the individual level, e.g.: oral chlorhexidine is a common omission in ICUs, but, in the ICUs using chlorhexidine, omission is rare at the individual)   ( x ) Yes ( ) No |
| 1. Can we generate an objective question (recommendation) associated with a clear intervention?   ( x ) Yes ( ) No |
| **Conclusion:** Should the item be included in the checklist?  ( x ) Yes ( ) No |

Reference List

1. Klevens RM, Edwards JR, Richards CL, Jr., Horan TC, Gaynes RP, Pollock DA, Cardo DM: **Estimating health care-associated infections and deaths in U.S. hospitals, 2002.** *Public Health Rep* 2007, **122:**160-166.

2. Daniels KR, Lee GC, Frei CR: **Trends in catheter-associated urinary tract infections among a national cohort of hospitalized adults, 2001-2010.** *Am J Infect Control* 2014, **42:**17-22.

3. Lo E, Nicolle L, Classen D, Arias KM, Podgorny K, Anderson DJ, Burstin H, Calfee DP, Coffin SE, Dubberke ER et al.: **Strategies to prevent catheter-associated urinary tract infections in acute care hospitals.** *Infect Control Hosp Epidemiol* 2008, **29 Suppl 1:**S41-S50.

4. Gould CV, Umscheid CA, Agarwal RK, Kuntz G, Pegues DA, and the Healthcare Infection Control Practices Advisory Committee (HICPAC). **Guideline for prevention of cateter-associated urinary tract infections 2009.** 2009. Healthcare Infection Control Practices Advisory Committee/Centers for Disease Control.

5. Schumm K, Lam TB: **Types of urethral catheters for management of short-term voiding problems in hospitalised adults.** *Cochrane Database Syst Rev* 2008,CD004013.

6. Murphy C, Fader M, Prieto J: **Interventions to minimise the initial use of indwelling urinary catheters in acute care: a systematic review.** *Int J Nurs Stud* 2014, **51:**4-13.

7. Meddings J, Rogers MA, Krein SL, Fakih MG, Olmsted RN, Saint S: **Reducing unnecessary urinary catheter use and other strategies to prevent catheter-associated urinary tract infection: an integrative review.** *BMJ Qual Saf* 2014, **23:**277-289.

Low-tidal volume ventilation for ARDS patients

**Clinical questions:**

Does low-tidal volume (≤6mL/kg) reduces mortality, length of ICU and hospital stay in ARDS patients compared to conventional tidal volumes (≥10 mL/kg)?

# Identify and critically appraise systematic reviews of randomized trials on the topic

## Low tidal volume for ARDS patients

We have identified a systematic review by Burns et al which assessed whether volume and pressure limited ventilation strategies are associated with better clinical outcomes than more traditional strategies.[17]

Ten trials were eligible for the systematic review, although in two trials liberal PEEP and alveolar recruitment maneuver were also applied for the experimental low-tidal volume group.[18,19]

Tidal volume recommendations in the experimental group varied between trials. ARDSNet trial recommended 4 to 6 mL/kg for predicted body weight (PBW). If there was severe acidosis or dyspnea, tidal volume could be increased up to 8 mL/Kg of PBW. Most other trials recommended tidal volumes between 4 and 8mL/kg in the experimental group, with two trial allowing up to 10 mL/kg.

Randomization was concealed in 8 of 10 trials; potential for selective outcome reporting bias in 5 of 10 trials; early stopping for benefit in 3 trials (for example the large ARDSNet trial) or futility in 3 trials. Blinding was not possible in all trials.

Pooled effect on mortality is shown in figure 1 (reproduced from Burns et al).[17] If we consider the trials comparing low-tidal volume to conventional ventilation excluding trials associating alveolar recruitment and high PEEP in the conventional group, the pooled relative risk for hospital mortality was 0.9, with 95%CI including beneficial, neutral and slightly harmful effect (95%CI 0.74 to 1.09). Also, heterogeneity was moderate, with two stopped early trials showing larger effects (ARDSNet trial and Wu).[20,21]

Use of neuromuscular blocking agents was more frequent with the low-tidal volume strategy, although with substantial inconsistency between trials (Relative risk 1.37; 95%CI 1.04 to 1.82; I^2^=59%).

**Figure 1.** Meta-analysis of randomized trials assessing the effect of pressure and volume limited ventilation versus conventional ventilation on in-hospital mortality

**Table 1.** Low tidal volume for ARDS: Summary of findings table with GRADE assessment of quality of evidence*

| **Quality assessment** | | | | | | | **Summary of findings** | | | | | **Importance** |
| --- | --- | --- | --- | --- | --- | --- | --- | --- | --- | --- | --- | --- |
|  |  |  |  |  |  |  | **No of patients** | | **Effect** | | **Quality** |  |
| **No of studies** | **Design** | **Limitations** | **Inconsistency** | **Indirectness** | **Imprecision** | **Publication bias** | **Low tidal volume** | **Control** | **Relative (95% CI)** | **Absolute** |  |  |
| **In-hospital Mortality** | | | | | | | | | | | | |
| 8 | Randomised trials | No serious limitations^1^ | No serious inconsistency^2^ | No serious indirectness | Serious^2^ | Unlikely | 282/809  (34.9%) | 324/792 (40.9%) | RR 0.90  (0.74 to 1.09) | 61 fewer deaths per 1000 patients (from 106 fewer to 37 more deaths per 1000) | ⊕⊕⊕O MODERATE (to LOW) | Critical |
|  | | | | | | | | | | | | |

* Studies of low tidal volume vs higher tidal volume for ARDS with additional co-interventions in the low-tidal volume group were not considered here.

1. Randomization was concealed in 8 of 10 trials; early stopping for benefit in 3 trials (for example the large ARDSNet trial) or futility in 3 trials. Blinding was not possible in all trials.
2. Inconsistency is moderate, with I^2^=44.8%.
3. Imprecision is substantial, as 95%CI is compatible with both substantial benefit or slight harm.

# Strength of recommendation

**Table 2.** Considerations to determine the strength of recommendation

| Factor | Comment |
| --- | --- |
| Balance between desirable and undesirable effects | Mortality is potentially reduced with low-tidal volume ventilation. Other relevant clinical outcomes were reported inconsistently in primary studies precluding meta-analyses. |
| Quality of evidence | Moderate to low quality |
| Values and preferences | Mortality reduction clearly important for patients and relatives. |
| Costs (resource allocation) | Intervention has no cost and the potential to save money. |

We recommend (strong recommendation) that low tidal-volume (between 4 and 6mL/kg of predicted body weigth) and low plateau pressures (<30cmH_2_O) be applied for ARDS patients. Higher breath rates (up to 35 breaths per minute may be needed to avoid respiratory acidosis). If severe acidosis (pH<7.15) persists with high respiratory rate, tidal volume may be increased up to 8mL/kg PBW.

**Table 3.** Criteria to include item “Low tidal volume for patients with ARDS” in the daily round checklist

| 1. What is the relevance of the outcome(s) affected by the checklist item?  - In-hospital mortality   ( x ) Critical [e.g.: death] ( ) Important ( ) Moderate [e.g.: pressure ulcer] |
| --- |
| 1. Is the recommendation strong? Consider the determinants of the strength of recommendation:    1. Level of evidence (GRADE: risk of bias, inconsistency, inaccuracy, indirect evidence, publication bias)   ( ) High ( x ) Moderate ( ) Low ( ) Very Low   - 1. Is the balance between desirable and undesirable effects (adverse events and discomfort) favorable?   ( ) Highly favorable  ( x) Advantages in general higher than disadvantages  ( ) Close balance of advantages and disadvantages   - 1. Costs (allocation of resources: training, human resources [complex interventions], financial resources)   ( ) High ( x ) Low   - 1. Variability (or uncertainty) in the values ​​and preferences   ( ) High ( x ) Low  Based on the above mentioned considerations, the strength of recommendation is:  (x ) Strong ( ) Weak |
| 1. Is it applicable to most ICU patients?   ( ) All [100%] ( ) Many [30 to <100%] ( x ) Few [<30%] |
| 1. Are complications common, serious and costly?   ( ) Meets three criteria ( x ) Two criteria ( ) One or less |
| 1. Is omission common? (at the individual level, e.g.: oral chlorhexidine is a common omission in ICUs, but, in the ICUs using chlorhexidine, omission is rare at the individual)   ( x ) Yes ( ) No |
| 1. Can we generate an objective question (recommendation) associated with a clear intervention?   ( x ) Yes ( ) No |
| **Conclusion:** Should the item be included in the checklist?  ( x ) Yes ( ) No |

Low-tidal volume ventilation for patients without ARDS needing mechanical ventilation

**Clinical questions:**

Does low-tidal volume (<8mL/Kg) reduces mortality, length of ICU and hospital stay for patients without ARDS needing mechanical ventilation compared to conventional tidal volumes (>10 mL/kg)?

# Identify and critically appraise systematic reviews of randomized trials on the topic

## Low tidal volume for patients without ARDS needing mechanical ventilation

A systematic review with meta-analysis assessed the effect of low-tidal volume ventilation compared to conventional ventilation for patients without ARDS.[22] Twenty studies were considered in the review, 15 were randomized controlled trials and 5 were observational studies. Follow up was very short in most studies (median follow up only 21 hours). The setting was intensive care unit in only two from the 15 RCT, with all the remaining trials assessing low-tidal volume effect during surgery.

Randomization was concealed in 11 of 15 randomized controlled trials included, and follow-up was excellent with minimal loss. Limitations included a lack of blinding (all trials), a lack of intention-to treat analysis (12 trials), and early stopping for benefit (1 trial).

Pooled risk ratios suggest reduction in mortality (RR 0.64; 95%CI 0.46-0.86; I^2^=0%), lung injury (RR 0.33; 95%CI 0.23-0.47; I^2^=0%) and pulmonary infection (RR 0.52; 95%CI 0.33-0.82; I^2^=0%). However, observational studies contribute with most of the weight in meta-analyses. Considering only randomized trials, the pooled effect estimate did not suggest a reduction in mortality (RR 0.97; 95%CI 0.53-1.78). Although, there may be decreased risk of lung injury (RR 0.26; 95%CI 0.10-0.66) and pulmonary infection (RR 0.27; 95%CI 0.12-0.64).

**Figure 1.** Meta-analysis of randomized trials assessing the effect of low tidal volume ventilation versus conventional tidal volume on mortality

**Figure 2.** Meta-analysis of randomized trials assessing the effect of low tidal volume ventilation versus conventional tidal volume on lung injury

**Figure 3.** Meta-analysis of randomized trials assessing the effect of low tidal volume ventilation versus conventional tidal volume on pulmonary infection

**Table 1.** Low tidal volume for patients without ARDS: Summary of findings table with GRADE assessment of quality of evidence*

| **Quality assessment** | | | | | | | **Summary of findings** | | | | | **Importance** |
| --- | --- | --- | --- | --- | --- | --- | --- | --- | --- | --- | --- | --- |
|  |  |  |  |  |  |  | **No of patients** | | **Effect** | | **Quality** |  |
| **No of studies** | **Design** | **Limitations** | **Inconsistency** | **Indirectness** | **Imprecision** | **Publication bias** | **Low tidal volume** | **Control** | **Relative (95% CI)** | **Absolute** |  |  |
| **Mortality** | | | | | | | | | | | | |
| 5 | Randomised trials | No serious limitations | No | Serious indirectness^1^ | Serious^2^ | Unlikely | 28/247  (11.3) | 28/244  (11.4) | RR 0.97  (0.53-1.78) | 1 fewer deaths per 1000 patients (from 54 fewer to 90 more deaths per 1000 | ⊕⊕OO LOW | Critical |
| **Lung injury** | | | | | | | | | | | | |
| 4 | Randomised trials | No serious limitations | No | No | Serious^3^ | Unlikely | 6/172  (3.5) | 21/170  (12.4) | RR 0.26  (0.10-0.66) | 89 fewer lung injuries per 1000 patients (from 111 to 42 fewer per 1000 patients) | ⊕⊕⊕O Moderate | Important |
| **Pulmonary infection** | | | | | | | | | | | | |
| 3 | Randomized trials | No serious limitations | No | No | Serious^3^ | Unlikely | 9/123  (7.3) | 27/132  (20.5) | RR 0.27  (0.12-0.64) | 131 fewer lung injuries per 1000 patients (from 180 to 74 fewer per 1000 patients) | ⊕⊕⊕O Moderate | Important |

* Only randomized trials included in Serpa-Neto[22] systematic review are considered here.

1. Data are mostly from trials performed on the surgical block. Only 2 from the 15 trials were conducted in the ICU.
2. Imprecision is substantial, as 95%CI is compatible with both substantial benefit or harm.
3. Few events observed (27 lung injuries and 36 pulmonary infections) makes effect estimates quite susceptible to change with accumulating data

**Table 2.** Criteria to include item “Low tidal volume for patients without ARDS” in the daily round checklist

| 1. What is the relevance of the outcome(s) affected by the checklist item?  - Reduction of lung injury and pulmonary infection   ( ) Critical [e.g.: death] ( x ) Important ( ) Moderate [e.g.: pressure ulcer] |
| --- |
| 1. Is the recommendation strong? Consider the determinants of the strength of recommendation:    1. Level of evidence (GRADE: risk of bias, inconsistency, inaccuracy, indirect evidence, publication bias)   ( ) High ( x ) Moderate ( ) Low ( ) Very Low   - 1. Is the balance between desirable and undesirable effects (adverse events and discomfort) favorable?   ( ) Highly favorable  ( x) Advantages in general higher than disadvantages  ( ) Close balance of advantages and disadvantages   - 1. Costs (allocation of resources: training, human resources [complex interventions], financial resources)   ( ) High ( x ) Low   - 1. Variability (or uncertainty) in the values ​​and preferences   ( ) High ( x ) Low  Based on the above mentioned considerations, the strength of recommendation is:  ( ) Strong ( x ) Weak |
| 1. Is it applicable to most ICU patients?   ( ) All [100%] ( x ) Many [30 to <100%] ( x ) Few [<30%] |
| 1. Are complications common, serious and costly?   ( x ) Meets three criteria ( ) Two criteria ( ) One or less |
| 1. Is omission common? (at the individual level, e.g.: oral chlorhexidine is a common omission in ICUs, but, in the ICUs using chlorhexidine, omission is rare at the individual)   ( x ) Yes ( ) No |
| 1. Can we generate an objective question (recommendation) associated with a clear intervention?   ( x ) Yes ( ) No |
| **Conclusion:** Should the item be included in the checklist?  ( x ) Yes ( ) No |

Reference List

1. Burns KE, Adhikari NK, Slutsky AS, Guyatt GH, Villar J, Zhang H, Zhou Q, Cook DJ, Stewart TE, Meade MO. Pressure and volume limited ventilation for the ventilatory management of patients with acute lung injury: a systematic review and meta-analysis. PLoS One. 2011; 6:e14623.

2. Amato MB, Barbas CS, Medeiros DM, Magaldi RB, Schettino GP, Lorenzi-Filho G, Kairalla RA, Deheinzelin D, Munoz C, Oliveira R, Takagaki TY, Carvalho CR. Effect of a protective-ventilation strategy on mortality in the acute respiratory distress syndrome. N Engl J Med. 1998; 338:347-354.

3. Villar J, Kacmarek RM, Perez-Mendez L, Aguirre-Jaime A. A high positive end-expiratory pressure, low tidal volume ventilatory strategy improves outcome in persistent acute respiratory distress syndrome: a randomized, controlled trial. Crit Care Med. 2006; 34:1311-1318.

4. Ventilation with lower tidal volumes as compared with traditional tidal volumes for acute lung injury and the acute respiratory distress syndrome. The Acute Respiratory Distress Syndrome Network. N Engl J Med. 2000; 342:1301-1308.

5. Wu G, Lu B. The application of low tidal volume pressure-controlled ventilation in patients with acute respiratory distress syndrome. Bull Hunan Med Univ. 1998; 23:57-58.

6. Serpa-Neto A, Cardoso SO, Manetta JA, Pereira VG, Esposito DC, Pasqualucci MO, Damasceno MC, Schultz MJ. Association between use of lung-protective ventilation with lower tidal volumes and clinical outcomes among patients without acute respiratory distress syndrome: a meta-analysis. JAMA. 2012; 308:1651-1659.

Semirecumbent position

**Clinical question: Does semirecumbent position (≥30°) reduces the risk of ventilator-associated pneumonia and mortality compared to a neutral position in critically ill patients?**

# Identify and critically appraise systematic reviews of randomized trials on the topic

We have found a systematic review of randomized controlled trials by Niël-Weisse et al. published in 2011.[1] Three RCT were included in this review.

The trial by Drakulovic et al. assessed 45° head-of-bed (HOB) position in 39 patients versus 0° HOB position in 47 adult critically-ill patients.[2] Allocation concealment was adequate and there was no evidence of selective outcome reporting. However, event assessors were not blind to treatment assignment and the study stopped early for benefit with a small number of events, potentially overestimating treatment effect.

The trial by Keeley et al. analysed the effect of 45° HOB position versus 25° HOB position in adult mechanically ventilated patients.[3] Only 30 patients were analysed, with 3 events of VAP. Although randomization was concealed and there was no evidence of selective outcome reporting, event assessors were not blinded and 46% of randomized patients were not included in analyses.

The larger trial, by Nieuwenhoven et al, enrolled 221 adult mechanically ventilated patients, who were assigned 45° versus 10° HOB position.[4] This is the only one from the three RCT with low risk of bias, as it reported adequate concealed randomization, no evidence of selective outcome reporting, blinding of outcome assessors, adequate follow-up of patients and intention-to-treat analysis.

Pooled effects of 45° HOB position versus control on clinically suspected VAP and mortality are shown in figures 1 and 2. Table 1 displays a summary of findings with assessment of quality of evidence following the GRADE system.

Compared to a more neutral position (0°, 10° or 25°), a 45° HOB position was associated with half the odds of VAP, however with a wide confidence interval overlapping null effect (OR 0.47; 95%CI 0.19 to 1.17). Also, risk of bias was considerable, particularly in the two trials with lower odds ratio. Thus, we downgraded quality of evidence to low level due to elevated risk of bias from primary studies and imprecision. Pooled point estimate of the semirecumbent position effect on mortality was close to null effect, also with wide effect. Quality of evidence is low due to imprecision and substantial risk of bias.

Available randomized trials did not report effects on adverse events potentially affected by a 45° decubitus, such as hemodynamic instability or venous thromboembolism. Furthermore, there is no evidence from randomized trials on the effect of the commonly recommended and used 30° HOB position. Thus, current evidence is even weaker for 30° HOB due to indirectness, that is, there is only very low quality evidence supporting the effect of 30°HOB for preventing VAP.

**Figure 1.** Meta-analysis of randomized controlled trials of semirecumbent position: outcome clinically suspected ventilator-associated pneumonia

*Reproduced from Niël-Weisse et al.

**Figure 2.** Meta-analysis of randomized controlled trials of semirecumbent position: mortality

*Reproduced from Niël-Weisse et al.

| **Quality assessment** | | | | | | | **Summary of findings** | | | | | **Importance** |
| --- | --- | --- | --- | --- | --- | --- | --- | --- | --- | --- | --- | --- |
|  |  |  |  |  |  |  | **No of patients** | | **Effect** | | **Quality** |  |
| **No of studies** | **Design** | **Limitations** | **Inconsistency** | **Indirectness** | **Imprecision** | **Publication bias** | **Semirecumbent position** | **Control** | **Relative (95% CI)** | **Absolute** |  |  |
| **Clinically suspected ventilator-associated pneumonia** | | | | | | | | | | | | |
| 3 | Randomised trials | Serious^1^ | No serious inconsistency | No serious indirectness^2^ | Serious^3^ | Unlikely | 20/168 (11.9%) | 38/169 (22.5%) | RR 0.47  (0.19 to 1.17) | 106 fewer VAPs per 1000 (from 182 fewer to 38 more VAP per 1000) | ⊕⊕OO LOW | Important |
| **Mortality** | | | | | | | | | | | | |
| 3 | Randomised trials | Serious^1^ | No serious inconsistency | No serious indirectness | Serious^3^ | Unlikely | 45/168 (26.8%) | 50/169 (29.6) | RR 0.90  (0.64 to 1.27) | 28 fewer deaths per 1000 (from 107 fewer to 80 more deaths per 1000) | ⊕⊕OO LOW | Critical |

**Table 1.** Summary of findings table with GRADE assessment of quality of evidence

1. Serious limitations in primary studies with considerable risk of bias: blind assessment of VAP and intention-to-treat analysis in only one trial (v. Nieuwenhoven 2006); one trial stopped early for benefit (Drakulovic 1999); in one trial data from 46% of the patients randomized was not included in analysis.
2. Niël-Weise et al. were uncertain regarding directness of evidence, as one of the trials (Drakulovic 1999) head-of-bed was 0° in the control group, what they considered not to be standard care. However, the two other trials assessed 25° (Keeley 2007) and 10° (v. Nieuwenhoven 2006). Furthermore, there is neither good evidence of benefit for other intermediate positions (>0° and <45°) nor are these intermediate position standard of care. Thus, we do not consider this meta-analysis faces problems of indirectness.
3. There is substantial imprecision in effect estimates, as 95% CI is compatible with both beneficial and harmful effects.

# Strength of recommendation

**Table 2.** Considerations to determine the strength of recommendation

| Factor | Comment |
| --- | --- |
| Balance between desirable and undesirable effects | It is possible that a 45°HOB position decreases the likelihood of clinical VAP (evidence of low quality). Effect on mortality is uncertain. Adverse events were not reported in RCT, so a pooled estimate is not possible.  However, keeping patient in a 45° decubitus is not practical, interferes with frequent changing of positioning to prevent decubitus ulcers and may conflict with frequent ICU procedures. It is absolutely contraindicated for patients with recent thoracic or lumbar surgery, and relatively contraindicated is some instances such as pressure ulcers and hemodynamic instability. |
| Quality of evidence | Low-quality evidence suggests reduction in VAP and close to null effect on mortality with 45° HOB position. |
| Values and preferences | We assume that most patients and families regard prevention of VAP and its consequences as very important. |
| Costs (resource allocation) | Intervention has no cost and the potential to save money. |

We suggest (weak recommendation) that head of the bed be maintained between 30° to 45° for mechanically ventilated patients. Other patients at high risk of pneumonia, for example patients receiving enteral feeding, may also benefit from a semirecumbent position.

**Table 3.** Criteria to include item “Semirecumbent position” in the daily round checklist

| 1. What is the relevance of the outcome(s) affected by the checklist item?  - Possible reduction of ventilator-associated pneumonia. No effect on mortality   ( ) Critical [e.g.: death] ( x ) Important ( ) Moderate [e.g.: pressure ulcer] |
| --- |
| 1. Is the recommendation strong? Consider the determinants of the strength of recommendation:    1. Level of evidence (GRADE: risk of bias, inconsistency, inaccuracy, indirect evidence, publication bias)   ( ) High ( ) Moderate ( x ) Low ( ) Very Low   - 1. Is the balance between desirable and undesirable effects (adverse events and discomfort) favorable?   ( ) Highly favorable  ( x) Advantages in general higher than disadvantages  ( ) Close balance of advantages and disadvantages   - 1. Costs (allocation of resources: training, human resources [complex interventions], financial resources)   ( ) High ( x ) Low   - 1. Variability (or uncertainty) in the values ​​and preferences   ( ) High ( x ) Low  Based on the above mentioned considerations, the strength of recommendation is:  ( ) Strong ( x ) Weak |
| 1. Is it applicable to most ICU patients?   ( ) All [100%] ( x ) Many [30 to <100%] ( ) Few [<30%] |
| 1. Are complications common, serious and costly?   ( x ) Meets three criteria ( ) Two criteria ( ) One or less |
| 1. Is omission common? (at the individual level, e.g.: oral chlorhexidine is a common omission in ICUs, but, in the ICUs using chlorhexidine, omission is rare at the individual)   ( x ) Yes ( ) No |
| 1. Can we generate an objective question (recommendation) associated with a clear intervention?   ( x ) Yes ( ) No |
| **Conclusion:** Should the item be included in the checklist?  ( x ) Yes ( ) No |

Reference List

1. Niel-Weise BS, Gastmeier P, Kola A, Vonberg RP, Wille JC, van den Broek PJ: **An evidence-based recommendation on bed head elevation for mechanically ventilated patients.** *Crit Care* 2011, **15:**R111.

2. Drakulovic MB, Torres A, Bauer TT, Nicolas JM, Nogue S, Ferrer M: **Supine body position as a risk factor for nosocomial pneumonia in mechanically ventilated patients: a randomised trial.** *Lancet* 1999, **354:**1851-1858.

3. Keeley L: **Reducing the risk of ventilator-acquired pneumonia through head of bed elevation.** *Nurs Crit Care* 2007, **12:**287-294.

4. van Nieuwenhoven CA, Vandenbroucke-Grauls C, van Tiel FH, Joore HC, van Schijndel RJ, van dT, I, Ramsay G, Bonten MJ: **Feasibility and effects of the semirecumbent position to prevent ventilator-associated pneumonia: a randomized study.** *Crit Care Med* 2006, **34:**396-402.

Pain control in the ICU

**Clinical question: Should monitoring and control of pain be systematically implemented in the ICU and how? Does it improve outcomes?**

# Identify and critically appraise systematic reviews of randomized trials on the topic

Pain management has been identified as a fundamental human right. Yet, despite significant advances in pain control after the gate control theory of pain was introduced in 1965, patients continue to experience pain[1]. Pain in intensive care unit (ICU) patients has received considerable attention in the last 20 years. Unrelieved pain has long been identified as one of the greatest concerns for ICU patients, can cause insufficient sleep, and is one of the main sources of psychological stress for ICU patients[2]. Pain associated with stress can persist after hospital discharge, adding to a long-term psychological burden on patients[3].

Studies demonstrate and guidelines endorse the knowledge that pain is frequent, and often neglected, in the critically ill (medical and surgical), especially those undergoing mechanical ventilation[4,5]. Pain control is associated with less use of sedatives, shorter ICU length of stay (in surgical patients), shorter duration of mechanical ventilation in a before-after study [6]. Albeit no studies demonstrated reduction in mortality, it is associated with less cardiovascular stress and systemic inflammatory response[7].

Pain itself is an important patient-centered outcome. We did not find systemtic reviews assessing the effects of opioids or other analgesics on pain control in critically ill patients. However, there are clear evidence from patient observation that opioids have a substantial effect on pain control, with a fast start of action and a dose-response effect. Thus, we have considered that these drugs are effective for pain control with a high quality of evidence.

Furthermore, a before-after study and a systematic review of acute pain management suggest that these strategies are safe for inpatients[8,9].

# Strength of recommendation

**Table 1.** Considerations to determine the strength of recommendation

| Factor | Comment |
| --- | --- |
| Balance between desirable and undesirable effects | Opioids effectively reduce acute pain in critically ill patients, contribute to control anxiety and reduce sedatives dose.  Adverse effects are well known and adequate acute pain management strategies have a low incidence of adverse effect. |
| Quality of evidence | High quality evidence that opioids effectively control pain.  Low-quality evidence suggests control of pain improves outcomes of critically ill (especially those under MV) |
| Values and preferences | Most patients and families regard control of pain and its consequences as very important. |
| Costs (resource allocation) | Intervention has low cost and the potential to save money. |

We suggest (weak recommendation) that validated scales are used to screen for pain every 1-2h and prompt control of pain and use of preprocedural pain control should be implemented for all ICU patients[4,10].

**Table 2.** Criteria to include pain assessment and control in the daily round checklist

| 1. What is the relevance of the outcome(s) affected by the checklist item?  - Pain reduction   ( ) Critical [e.g.: death] ( ) Important ( x ) Moderate [e.g.: pressure ulcer] |
| --- |
| 1. Is the recommendation strong? Consider the determinants of the strength of recommendation:    1. Level of evidence (GRADE: risk of bias, inconsistency, inaccuracy, indirect evidence, publication bias)   ( x ) High ( ) Moderate ( ) Low ( ) Very Low   - 1. Is the balance between desirable and undesirable effects (adverse events and discomfort) favorable?   ( ) Highly favorable  ( x) Advantages in general higher than disadvantages  ( ) Close balance of advantages and disadvantages   - 1. Costs (allocation of resources: training, human resources [complex interventions], financial resources)   ( ) High ( x ) Low   - 1. Variability (or uncertainty) in the values ​​and preferences   ( ) High ( x ) Low  Based on the above mentioned considerations, the strength of recommendation is:  (x ) Strong ( ) Weak |
| 1. Is it applicable to most ICU patients?   ( ) All [100%] ( x ) Many [30 to <100%] ( x ) Few [<30%] |
| 1. Are complications common, serious and costly?   ( ) Meets three criteria ( x ) Two criteria ( ) One or less |
| 1. Is omission common? (at the individual level, e.g.: oral chlorhexidine is a common omission in ICUs, but, in the ICUs using chlorhexidine, omission is rare at the individual)   ( x ) Yes ( ) No |
| 1. Can we generate an objective question (recommendation) associated with a clear intervention?   ( x ) Yes ( ) No |
| **Conclusion:** Should the item be included in the checklist?  ( x ) Yes ( ) No |

Reference List

1. Melzack R, Wall PD: **Pain mechanisms: a new theory.** *Science* 1965, **150:**971-979.

2. Jones J, Hoggart B, Withey J, Donaghue K, Ellis BW: **What the patients say: A study of reactions to an intensive care unit.** *Intensive Care Med* 1979, **5:**89-92.

3. Granja C, Gomes E, Amaro A, Ribeiro O, Jones C, Carneiro A, Costa-Pereira A: **Understanding posttraumatic stress disorder-related symptoms after critical care: the early illness amnesia hypothesis.** *Crit Care Med* 2008, **36:**2801-2809.

4. Barr J, Fraser GL, Puntillo K, Ely EW, Gelinas C, Dasta JF, Davidson JE, Devlin JW, Kress JP, Joffe AM et al.: **Clinical practice guidelines for the management of pain, agitation, and delirium in adult patients in the intensive care unit.** *Crit Care Med* 2013, **41:**263-306.

5. Puntillo KA, Max A, Timsit JF, Vignoud L, Chanques G, Robleda G, Roche-Campo F, Mancebo J, Divatia JV, Soares M et al.: **Determinants of procedural pain intensity in the intensive care unit. The Europain(R) study.** *Am J Respir Crit Care Med* 2014, **189:**39-47.

6. Chanques G, Jaber S, Barbotte E, Violet S, Sebbane M, Perrigault PF, Mann C, Lefrant JY, Eledjam JJ: **Impact of systematic evaluation of pain and agitation in an intensive care unit.** *Crit Care Med* 2006, **34:**1691-1699.

7. Akca O, Melischek M, Scheck T, Hellwagner K, Arkilic CF, Kurz A, Kapral S, Heinz T, Lackner FX, Sessler DI: **Postoperative pain and subcutaneous oxygen tension.** *Lancet* 1999, **354:**41-42.

8. Frasco PE, Sprung J, Trentman TL: **The impact of the joint commission for accreditation of healthcare organizations pain initiative on perioperative opiate consumption and recovery room length of stay.** *Anesth Analg* 2005, **100:**162-168.

9. Helfand M, Freeman M: **Assessment and management of acute pain in adult medical inpatients: a systematic review.** *Pain Med* 2009, **10:**1183-1199.

10. Gelinas C, Puntillo KA, Joffe AM, Barr J: **A validated approach to evaluating psychometric properties of pain assessment tools for use in nonverbal critically ill adults.** *Semin Respir Crit Care Med* 2013, **34:**153-168.

Light sedation

**Clinical question:** Should adult ICU patients be maintained at a light level of sedation?

# Identify and critically appraise systematic reviews of randomized trials on the topic

Four randomized controlled trials demonstrate that light levels of sedation are associated with shorter duration of mechanical ventilation and length of ICU stay [1-4]. We have found no systematic review with meta-analysis on this topic. The Clinical Practice Guidelines for the Management of Pain, Agitation, and Delirium in Adult Patients in the Intensive Care Unit considered the quality of evidence as moderate[5].

It is unclear whether daily sedation interruption (DSI) offers any advantage over no daily interruption [6]. In fact, a randomized trial comparing DSI with protocolized light sedation found no difference in length of ICU stay or duration of mechanical ventilation. However, in the DSI group there was increased use of sedatives and opioids, both as mean daily doses or need of boluses, and increased nursing workload [7].

Maintaining light levels of sedation increase physiologic response to stress, but there is no increase in myocardial ischemia [8,9].

# Strength of recommendation

**Table 1.** Considerations to determine the strength of recommendation

| Factor | Comment |
| --- | --- |
| Balance between desirable and undesirable effects | Evidence from randomized trials demonstrate benefit in terms of reduced LOS in ICU and reduced mechanical ventilation. Some studies also suggest lower incidence of post-traumatic stress syndrome.  There is increase in physiologic stress response, although with no evidence of deleterious effect to patients.  There RCTs suggest adverse events are not increased in patients under light sedation. |
| Quality of evidence | Moderate (effect on length of ICU stay and duration of mechanical ventilation). |
| Values and preferences | Shorter length of stay in ICU is important for patients and relatives. |
| Costs (resource allocation) | Intervention has low cost and the potential to save money. |

We consider that the strength of recommendation is strong.

**Table 2.** Criteria to include light sedation in the daily round checklist

| 1. What is the relevance of the outcome(s) affected by the checklist item?  - Length of stay in ICU and shorter MV duration   ( ) Critical [e.g.: death] ( x ) Important ( ) Moderate [e.g.: pressure ulcer] |
| --- |
| 1. Is the recommendation strong? Consider the determinants of the strength of recommendation:    1. Level of evidence (GRADE: risk of bias, inconsistency, inaccuracy, indirect evidence, publication bias)   ( ) High ( x ) Moderate ( ) Low ( ) Very Low   - 1. Is the balance between desirable and undesirable effects (adverse events and discomfort) favorable?   ( ) Highly favorable  ( x) Advantages in general higher than disadvantages  ( ) Close balance of advantages and disadvantages   - 1. Costs (allocation of resources: training, human resources [complex interventions], financial resources)   ( ) High ( x ) Low   - 1. Variability (or uncertainty) in the values ​​and preferences   ( ) High ( x ) Low  Based on the above mentioned considerations, the strength of recommendation is:  (x ) Strong ( ) Weak |
| 1. Is it applicable to most ICU patients?   ( ) All [100%] ( x ) Many [30 to <100%] ( x ) Few [<30%] |
| 1. Are complications common, serious and costly?   ( ) Meets three criteria ( x ) Two criteria ( ) One or less |
| 1. Is omission common? (at the individual level, e.g.: oral chlorhexidine is a common omission in ICUs, but, in the ICUs using chlorhexidine, omission is rare at the individual)   ( x ) Yes ( ) No |
| 1. Can we generate an objective question (recommendation) associated with a clear intervention?   ( x ) Yes ( ) No |
| **Conclusion:** Should the item be included in the checklist?  ( x ) Yes ( ) No |

Reference List

1. Brook AD, Ahrens TS, Schaiff R, Prentice D, Sherman G, Shannon W, Kollef MH: **Effect of a nursing-implemented sedation protocol on the duration of mechanical ventilation.** *Crit Care Med* 1999, **27:**2609-2615.

2. Girard TD, Kress JP, Fuchs BD, Thomason JW, Schweickert WD, Pun BT, Taichman DB, Dunn JG, Pohlman AS, Kinniry PA et al.: **Efficacy and safety of a paired sedation and ventilator weaning protocol for mechanically ventilated patients in intensive care (Awakening and Breathing Controlled trial): a randomised controlled trial.** *Lancet* 2008, **371:**126-134.

3. Kress JP, Pohlman AS, O'Connor MF, Hall JB: **Daily interruption of sedative infusions in critically ill patients undergoing mechanical ventilation.** *N Engl J Med* 2000, **342:**1471-1477.

4. Treggiari MM, Romand JA, Yanez ND, Deem SA, Goldberg J, Hudson L, Heidegger CP, Weiss NS: **Randomized trial of light versus deep sedation on mental health after critical illness.** *Crit Care Med* 2009, **37:**2527-2534.

5. Barr J, Fraser GL, Puntillo K, Ely EW, Gelinas C, Dasta JF, Davidson JE, Devlin JW, Kress JP, Joffe AM et al.: **Clinical practice guidelines for the management of pain, agitation, and delirium in adult patients in the intensive care unit.** *Crit Care Med* 2013, **41:**263-306.

6. Burry L, Rose L, McCullagh IJ, Fergusson DA, Ferguson ND, Mehta S: **Daily sedation interruption versus no daily sedation interruption for critically ill adult patients requiring invasive mechanical ventilation.** *Cochrane Database Syst Rev* 2014, **7:**CD009176.

7. Mehta S, Burry L, Cook D, Fergusson D, Steinberg M, Granton J, Herridge M, Ferguson N, Devlin J, Tanios M et al.: **Daily sedation interruption in mechanically ventilated critically ill patients cared for with a sedation protocol: a randomized controlled trial.** *JAMA* 2012, **308:**1985-1992.

8. Kress JP, Vinayak AG, Levitt J, Schweickert WD, Gehlbach BK, Zimmerman F, Pohlman AS, Hall JB: **Daily sedative interruption in mechanically ventilated patients at risk for coronary artery disease.** *Crit Care Med* 2007, **35:**365-371.

9. Hall RI, MacLaren C, Smith MS, McIntyre AJ, Allen CT, Murphy JT, Sullivan J, Wood J, Ali I, Kinley E: **Light versus heavy sedation after cardiac surgery: myocardial ischemia and the stress response. Maritime Heart Centre and Dalhousie University.** *Anesth Analg* 1997, **85:**971-978.

Discontinuation of mechanical ventilation

**Clinical questions:** Should we daily consider discontinuation of mechanical ventilation? Is protocolized weaning associated with better outcomes?

# Identify and critically appraise systematic reviews of randomized trials on the topic

Prolonged mechanical ventilation (MV) is associated with worse outcomes such as increased mortality[1] and incidence of pneumonia[2]. Protocols for discontinuation of mechanical ventilation in general include three components: objective criteria to assess readiness to wean; guidelines for reducing ventilator support, which in general include a spontaneous breathing trial, either on T-piece or pressure support; criteria to decide whether the patient is ready for extubation.

A systematic review of randomized controlled trials comparing protocolized weaning versus no protocolized weaning of mechanical ventilation suggested a 25% reduction in the mean duration of MV and 10% reduction in the length of stay in ICU[3]. However, authors considered the quality of evidence as low mainly due to substantial unexplained heterogeneity between trials’ results. Also, clinical settings, weaning protocol and control group care varied greatly between trials.

# Strength of recommendation

**Table 1.** Considerations to determine the strength of recommendation

| Factor | Comment |
| --- | --- |
| Balance between desirable and undesirable effects | Protocolized weaning probably reduces duration of MV and length of stay in ICU. No undesirable effects were reported. |
| Quality of evidence | Low |
| Values and preferences | As only desirable effects were reported, values and preferences of patients, families and clinicians clearly favor protocolized weaning. |
| Costs (resource allocation) | Three trials included in the meta-analysis assessed costs without statistically significant reduction in the protocolized weaning group. |

**Table 2.** Criteria to include assessment of discontinuations of mechanical ventilation in the daily round checklist

| 1. What is the relevance of the outcome(s) affected by the checklist item?  - Shorter MV duration and length of stay in ICU   ( ) Critical [e.g.: death] ( x ) Important ( ) Moderate [e.g.: pressure ulcer] |
| --- |
| 1. Is the recommendation strong? Consider the determinants of the strength of recommendation:    1. Level of evidence (GRADE: risk of bias, inconsistency, inaccuracy, indirect evidence, publication bias)   ( ) High ( ) Moderate ( x ) Low ( ) Very Low   - 1. Is the balance between desirable and undesirable effects (adverse events and discomfort) favorable?   ( x ) Highly favorable  ( ) Advantages in general higher than disadvantages  ( ) Close balance of advantages and disadvantages   - 1. Costs (allocation of resources: training, human resources [complex interventions], financial resources)   ( ) High ( x ) Low   - 1. Variability (or uncertainty) in the values ​​and preferences   ( ) High ( x ) Low  Based on the above mentioned considerations, the strength of recommendation is:  (x ) Strong ( ) Weak |
| 1. Is it applicable to most ICU patients?   ( ) All [100%] ( x ) Many [30 to <100%] ( x ) Few [<30%] |
| 1. Are complications common, serious and costly?   ( x ) Meets three criteria ( ) Two criteria ( ) One or less |
| 1. Is omission common? (at the individual level, e.g.: oral chlorhexidine is a common omission in ICUs, but, in the ICUs using chlorhexidine, omission is rare at the individual)   ( x ) Yes ( ) No |
| 1. Can we generate an objective question (recommendation) associated with a clear intervention?   ( x ) Yes ( ) No |
| **Conclusion:** Should the item be included in the checklist?  ( x ) Yes ( ) No |

**Reference List**

1. Dries DJ: **Weaning from mechanical ventilation.** *J Trauma* 1997, **43:**372-384.

2. Cook DJ, Walter SD, Cook RJ, Griffith LE, Guyatt GH, Leasa D, Jaeschke RZ, Brun-Buisson C: **Incidence of and risk factors for ventilator-associated pneumonia in critically ill patients.** *Ann Intern Med* 1998, **129:**433-440.

3. Blackwood B, Alderdice F, Burns K, Cardwell C, Lavery G, O'Halloran P: **Use of weaning protocols for reducing duration of mechanical ventilation in critically ill adult patients: Cochrane systematic review and meta-analysis.** *BMJ* 2011, **342:**c7237.

Oral hygiene with chlorhexidine

**Clinical question:** Does oral hygiene with chlrohexidine twice daily (or more frequent) reduce ventilator-associated pneumonia (VAP) and in-hospital mortality in mechanically ventilated patients?

# Identify and critically appraise systematic reviews of randomized trials on the topic

Oral hygiene with chlorhexidine has been adopted in most ICUs for mechanically ventilated patients based on results from influential systematic reviews and recommendations of guidelines [1-4]. In most ICUs these practice has been protocolized and it is included in nurses prescription.

In fact, several systematic reviews with meta-analysis of randomized trials assessed the effect of oral hygiene with chlorhexidine on ventilator-associated pneumonia and in-hospital mortality with conflicting results [1,4-11]. We commented below the most recent reviews.

Klompas et al. identified 16 randomized trials comparing oral chlorehexidine with inert solutiions[5]. This reviews found that within post-cardiac surgery patients, there was a beneficial effect of chlorhexidine on VAP (relative risk[RR] 0.56 [95%CI, 0.41-0.77]), but no effect for other critically ill patients (RR, 0.88 [95%CI, 0.66-1.16]). There was a neutral effect on mortality for post-cardiac surgery patients, and a trend towards increased mortality for other critically ill patients (RR, 1.13 [95%CI, 0.99-1.29]). The review by Silvestri et. al identified 23 trials also showed a reduction of VAP, but limited to surgical patients[6]. There was no effect on mortality. Finally, at odds with previows reviews, the review by Price et al. included 11 trials and showed that oral hygiene with chlorhexidine may increase mortality (odds ratio 1.25, 1.05 to 1.50).

We considered the quality of evidence as low due to substantial heterogeneity between trials’ results and elevated risk of bias in many trials.

The decision to adopt oral hygiene with chlorexidine is in general not left to individual doctors or nurses, but is rather a managerial decision which results in protocolized prescription of oral hygiene to all patients under MV by nurses. Thus, we consider that omission is uncommon at the patient level, which means that variation in this practice occur mainly between ICUs, but no between patients under MV on a given ICU.

# Strength of recommendation

**Table 1.** Considerations to determine the strength of recommendation

| Factor | Comment |
| --- | --- |
| Balance between desirable and undesirable effects | It is unclear whether chlorhexidine is effective in reducing VAP, mainly in clinical patients under MV.  Also, a recent systematic reviews raises issues regarding its safety. |
| Quality of evidence | Low quality (for both VAP and mortality) |
| Values and preferences | There is no conclusive evidence of effectiveness of oral hygiene with chlorhexidine on patient-centered outcomes, but one review which suggested harm (increased mortality). |
| Costs (resource allocation) | Low. |

We considered the strength of recommendation for using oral hygiene with chlorhexidine as weak.

| 1. What is the relevance of the outcome(s) affected by the checklist item?  - Reduction of ventilator-associated pneumonia   ( ) Critical [e.g.: death] ( ) Important (x ) Moderate [e.g.: pressure ulcer]   - Mortality   (x ) Critical [e.g.: death] ( ) Important ( ) Moderate [e.g.: pressure ulcer] |
| --- |
| 1. Is the recommendation strong? Consider the determinants of the strength of recommendation:    1. Level of evidence (GRADE: risk of bias, inconsistency, inaccuracy, indirect evidence, publication bias)   ( ) High ( x ) Moderate ( ) Low ( ) Very Low   - 1. Is the balance between desirable and undesirable effects (adverse events and discomfort) favorable?   ( ) Highly favorable  ( ) Advantages in general higher than disadvantages  ( x ) Close balance of advantages and disadvantages   - 1. Costs (allocation of resources: training, human resources [complex interventions], financial resources)   ( ) High ( x ) Low   - 1. Variability (or uncertainty) in the values ​​and preferences   ( x ) High ( ) Low  Based on the above mentioned considerations, the strength of recommendation is:  ( ) Strong ( x ) Weak |
| 1. Is it applicable to most ICU patients?   ( ) All [100%] ( x ) Many [30 to <100%] ( x ) Few [<30%] |
| 1. Are complications common, serious and costly?   ( ) Meets three criteria ( x ) Two criteria ( ) One or less |
| 1. Is omission common? (at the individual level, e.g.: oral chlorhexidine is a common omission in ICUs, but, in the ICUs using chlorhexidine, omission is rare at the individual)   ( ) Yes ( x ) No |
| 1. Can we generate an objective question (recommendation) associated with a clear intervention?   ( x ) Yes ( ) No |
| **Conclusion:** Should the item be included in the checklist?  ( ) Yes (x ) No |

**Table 1.** Criteria to include hygiene with oral chlorhexidine in the daily round checklist

Reference List

1. Labeau SO, Van d, V, Brusselaers N, Vogelaers D, Blot SI: **Prevention of ventilator-associated pneumonia with oral antiseptics: a systematic review and meta-analysis.** *Lancet Infect Dis* 2011, **11:**845-854.

2. Coffin SE, Klompas M, Classen D, Arias KM, Podgorny K, Anderson DJ, Burstin H, Calfee DP, Dubberke ER, Fraser V et al.: **Strategies to prevent ventilator-associated pneumonia in acute care hospitals.** *Infect Control Hosp Epidemiol* 2008, **29 Suppl 1:**S31-S40.

3. **How-to Guide: Prevent Ventilator-Associated Pneumonia.** 2012. Cambridge, MA, How-to Guide: Prevent Ventilator-Associated Pneumonia. Cambridge, MA.

4. Price R, MacLennan G, Glen J: **Selective digestive or oropharyngeal decontamination and topical oropharyngeal chlorhexidine for prevention of death in general intensive care: systematic review and network meta-analysis.** *BMJ* 2014, **348:**g2197.

5. Klompas M, Speck K, Howell MD, Greene LR, Berenholtz SM: **Reappraisal of routine oral care with chlorhexidine gluconate for patients receiving mechanical ventilation: systematic review and meta-analysis.** *JAMA Intern Med* 2014, **174:**751-761.

6. Silvestri L, Weir I, Gregori D, Taylor N, Zandstra D, Van Saene JJ, Van Saene HK: **Effectiveness of oral chlorhexidine on nosocomial pneumonia, causative micro-organisms and mortality in critically ill patients: a systematic review and meta-analysis.** *Minerva Anestesiol* 2014, **80:**805-820.

7. Zhang TT, Tang SS, Fu LJ: **The effectiveness of different concentrations of chlorhexidine for prevention of ventilator-associated pneumonia: a meta-analysis.** *J Clin Nurs* 2014, **23:**1461-1475.

8. Shi Z, Xie H, Wang P, Zhang Q, Wu Y, Chen E, Ng L, Worthington HV, Needleman I, Furness S: **Oral hygiene care for critically ill patients to prevent ventilator-associated pneumonia.** *Cochrane Database Syst Rev* 2013, **8:**CD008367.

9. Alhazzani W, Smith O, Muscedere J, Medd J, Cook D: **Toothbrushing for critically ill mechanically ventilated patients: a systematic review and meta-analysis of randomized trials evaluating ventilator-associated pneumonia.** *Crit Care Med* 2013, **41:**646-655.

10. Labeau SO, Van d, V, Brusselaers N, Vogelaers D, Blot SI: **Prevention of ventilator-associated pneumonia with oral antiseptics: a systematic review and meta-analysis.** *Lancet Infect Dis* 2011, **11:**845-854.

11. Chan EY, Ruest A, Meade MO, Cook DJ: **Oral decontamination for prevention of pneumonia in mechanically ventilated adults: systematic review and meta-analysis.** *BMJ* 2007, **334:**889.

Achieving optimal nutritional requirements

**Clinical question:** Achieving optimal individual nutritional requirements in ICU patients results in improved clinical outcomes in critically ill patients?

# Identify and critically appraise systematic reviews of randomized trials on the topic

We performed a literature review focusing primarily on studies aimed to evaluate the impact of protocolized care rather than in single nutritional interventions. We did a MEDLINE database search using the following descriptors: intensive care OR critical care OR ICU OR critically ill AND nutrition OR nutritional AND protocolized care OR quality improvement OR protocol implementation OR education OR performance using “systematic review” as a filter.

We only found a single systematic review, the Canadian guidelines, published in 2013 [1]. In this review, the authors stated that the previous guidelines from 2009 did not need updating as no other randomized controlled trial (RCT) was published in the subject. In the 2009 guidelines, there are three RCT on feeding protocols, but a meta-analysis was not performed [2-4]. Thus, we also did another search using the same descriptors above without the filter and adding the following new descriptors: randomized controlled trial [pt] OR controlled clinical trial [pt] OR randomized controlled trials [mh] OR random allocation [mh] OR double-blind method [mh] OR single-blind method [mh] OR clinical trial [pt] OR clinical trials [mh] OR "clinical trial" [tw]). We could find only one additional RCT [5].

It is worth mentioning that the American Society of Parenteral and Enteral Nutrition (ASPEN) also addressed this topic in their 2009 guidelines[6]. They recommend enteral feeding protocols based on the evidence from small randomized trials which suggest a increase in the overall percentage of goal calories provided, although clinical outcomes are not addressed.

The first RCT is a cluster randomized trial aiming to compare gastrointestinal tolerance of two enteral feeding protocols in critically ill patients so the control group also was protocolized [4].

The ACCEPT trial was also a cluster-randomized controlled trial comprising ICUs of 11 community and 3 teaching hospitals in Canada [5]. The primary outcomes were hospital mortality, length of ICU stay and length of hospital stay. They included patients at least 16 years of age with an expected ICU stay of at least 48 hours, 214 randomized to the control arm and 248 to the intervention one. The intervention was associated with more days of enteral nutrition (6.7 v. 5.4 per 10 patient-days; p = 0.042), shorter mean stay in hospital (25 v. 35 days; p = 0.003) and a trend toward reduced mortality (27% v. 37%; p = 0.058). They could not found any difference in ICU stay (10.9 v. 11.8 days; p = 0.7). Although they concluded that the implementation of evidence-based recommendations improved the provision of nutritional support and was associated with improved clinical outcomes, there are some issues in the study. Two hospitals crossed over and were excluded from the primary analysis. Also, they were not able to show significant differences in other relevant secondary outcomes as the total amount of energy delivered per patient-day, time from ICU admission to receiving enteral feeds, time required to achieve 80% of the calculated energy goal and the number of days on which 80% of the goal was achieved.

These clinical results were not confirmed in another cluster randomized trial [2]. The ANZICS group conducted this RCT in ICUs of 27 community and tertiary hospitals in Australia and New Zealand. They included 1118 critically ill adult patients expected to remain in the ICU longer than 2 days, 591 patients in the intervention arm and 557 in the control ICUs. Intervention ICUs fed patients earlier enteral (0.75 vs 1.37 mean days, difference, −0.62 [95% CI, −0.82 to −0.36]; P_.001) and parenterally (1.04 vs 1.40; difference, −0.35 [95% CI, −0.61 to −0.01]; P=.04) and also achieved caloric goals more often (6.10 vs 5.02 mean days per 10 fed patient-days; difference, 1.07 [95% CI, 0.12 to 2.22]; P=.03). However, there was no difference in hospital discharge mortality (28.9% vs 27.4%; difference, 1.4% [95% CI, −6.3% to 12.0%]; P=.75), hospital length of stay (24.2vs 24.3 days; difference, −0.08 [95% CI, −3.8 to 4.4]; P=.97) or ICU length of stay (9.1 vs 9.9 days; difference, −0.86 [95% CI, −2.6 to 1.3]; P=.42).

Another cluster RCT was not included in the Canadian guidelines [1]. Jain et al aimed to compare the passive mailing of the Canadian nutritional guidelines with a multifaceted educational intervention for implementation[5]. They included 58 ICUs in Canada and assessed 623 and 612 patients consecutive mechanically ventilated patients at each time period. The primary end point was nutritional adequacy of enteral nutrition. Although some changes in nutritional adequacy changed over time, these changes were similar in both groups (8.0% vs. 6.2 %, p=0.54). There were no differences in clinical outcomes between groups or across time periods.

The quality of evidence for the effect on mortality and other clinical outcomes is moderate as there is relevant imprecision in effect estimates both in ACCEPT and the ANZICS trial[2,3]. However, all clusterRCTs report improvements in nutritional outcomes such as time to start receiving diet or caloric delivery. Thus, we considered the quality of evidence for nutritional outcomes as high.

# Strength of recommendation

**Table 1.** Considerations to determine the strength of recommendation

| Factor | Comment |
| --- | --- |
| Balance between desirable and undesirable effects | There is substantial evidence that the implementation of feeding protocols is associated with improved nutrition outcomes like the amount of calories/protein delivery and days under nutrition. However, there is no clear evidence that improving nutrition support will lead to improved clinical outcomes.  Serious adverse events were not reported in these RCT, thus the likelihood of undesirable effects is low. |
| Quality of evidence | Moderate -quality evidence suggests no effect in clinical outcomes.  High quality evidence show beneficial effect on nutritional outcomes. |
| Values and preferences | We assume that optimization of nutritional support would be somewhat relevant. However, effects of important patient-centered outcomes were not demonstrated. |
| Costs (resource allocation) | Intervention has low cost but need resource allocation. |

**Table 2.** Criteria to include “Achieving optimal nutritional requirements” in the daily round checklist

| 1. What is the relevance of the outcome(s) affected by the checklist item?  - Mortality – no effect   ( x) Critical [e.g.: death] ( ) Important ( ) Moderate [e.g.: pressure ulcer]   - Nutritional goals (calories delivered, protein delivered, days under nutrition) - improved nutritional goals   ( ) Critical [e.g.: death] ( ) Important ( x) Moderate [e.g.: pressure ulcer] |
| --- |
| 1. Is the recommendation strong? Consider the determinants of the strength of recommendation:    1. Level of evidence (GRADE: risk of bias, inconsistency, inaccuracy, indirect evidence, publication bias) –No effect on mortality   ( ) High ( x ) Moderate ( ) Low ( ) Very Low   - 1. Is the balance between desirable and undesirable effects (adverse events and discomfort) favorable?   ( ) Highly favorable  ( ) Advantages in general higher than disadvantages  ( x ) Close balance of advantages and disadvantages   - 1. Costs (allocation of resources: training, human resources [complex interventions], financial resources)   ( x ) High ( ) Low   - 1. Variability (or uncertainty) in the values ​​and preferences   ( x ) High ( ) Low  Based on the above mentioned considerations, the strength of recommendation is:  ( ) Strong ( x ) Weak – against |
| 1. Is it applicable to most ICU patients?   ( ) All [100%] ( x ) Many [30 to <100%] ( x ) Few [<30%] |
| 1. Are complications common, serious and costly?   ( ) Meets three criteria ( ) Two criteria ( x ) One or less |
| 1. Is omission common? (at the individual level, e.g.: oral chlorhexidine is a common omission in ICUs, but, in the ICUs using chlorhexidine, omission is rare at the individual)   ( x ) Yes ( ) No |
| 1. Can we generate an objective question (recommendation) associated with a clear intervention?   ( x ) Yes ( ) No |
| **Conclusion:** Should the item be included in the checklist?*  ( ) Yes (x ) No |

*Although the proposition of the Steering Committee was against the inclusion of this item on the checklist, most investigators from the experimental ICUs voted for its inclusion in the Investigators’ Meeting. Thus, this item was included in the final version of the checklist.

Reference List

1. Dhaliwal R, Cahill N, Lemieux M, Heyland DK: **The Canadian critical care nutrition guidelines in 2013: an update on current recommendations and implementation strategies.** *Nutr Clin Pract* 2014, **29:**29-43.

2. Doig GS, Simpson F, Finfer S, Delaney A, Davies AR, Mitchell I, Dobb G: **Effect of evidence-based feeding guidelines on mortality of critically ill adults: a cluster randomized controlled trial.** *JAMA* 2008, **300:**2731-2741.

3. Martin CM, Doig GS, Heyland DK, Morrison T, Sibbald WJ: **Multicentre, cluster-randomized clinical trial of algorithms for critical-care enteral and parenteral therapy (ACCEPT).** *CMAJ* 2004, **170:**197-204.

4. Pinilla JC, Samphire J, Arnold C, Liu L, Thiessen B: **Comparison of gastrointestinal tolerance to two enteral feeding protocols in critically ill patients: a prospective, randomized controlled trial.** *JPEN J Parenter Enteral Nutr* 2001, **25:**81-86.

5. Jain MK, Heyland D, Dhaliwal R, Day AG, Drover J, Keefe L, Gelula M: **Dissemination of the Canadian clinical practice guidelines for nutrition support: results of a cluster randomized controlled trial.** *Crit Care Med* 2006, **34:**2362-2369.

6. McClave SA, Martindale RG, Vanek VW, McCarthy M, Roberts P, Taylor B, Ochoa JB, Napolitano L, Cresci G: **Guidelines for the Provision and Assessment of Nutrition Support Therapy in the Adult Critically Ill Patient: Society of Critical Care Medicine (SCCM) and American Society for Parenteral and Enteral Nutrition (A.S.P.E.N.).** *JPEN J Parenter Enteral Nutr* 2009, **33:**277-316.
