# Supplementary material for: A cluster randomized trial of a multifaceted quality improvement intervention in Brazilian intensive care units: study protocol
Source: Implement Sci. 2015 Jan 13;10:8. doi: 10.1186/s13012-014-0190-0 (PMC4342101; doi:10.1186/s13012-014-0190-0)
Supplement: Additional file 2: — CHECKLIST Elaboration. Step 3—Assessment of quality of evidence, strength of recommendation and criteria to include an item in the checklist. [file 13012_2014_190_MOESM2_ESM.docx]

**Additional file 2** Outcomes reflecting care processes

| **Outcome** | **Definition** | **When we assessed** | **How we assessed** |
| --- | --- | --- | --- |
| **Feeding** | $=\frac{number of patient-days receiving enteral or parenteral feeding}{patient-days}$ | Every 3 three days from ICU day 2 to 17. | Checked prescription |
| **Head of bed elevated at 30°** | $=\frac{number of patient-days with head of bed elevated at\geq30^{\circ}}{eligible patient-days}$ | Every 3 three days from ICU day 2 to 17. | Bed observation We considered only patients with indication of head of the bed at 30° (MV, enteral feeding or altered level of consciousness) |
| **Adequate prevention of venous thromboembolism** | $=\frac{number of patient-days with adequate DVT prevention}{patient-days}$  Adequate prevention of VTE is defined as: Dalteparin 5.000U 1x a day SC, enoxaparin 40mg 1x a day SC or heparin 5.000U 12/12hs SC or fondaparinux 2.5 mg 1x a day SC. A higher dose can be used if the patient has indication of anticoagulation therapy. If heparin is contraindicated: compression stockings ± pneumatic compression | Every 3 three days from ICU day 2 to 17. | Checked prescription |
| **Rate of antibiotic use** | $= \frac{number of patient-days with antibiotic prescription}{patient-days} X 100$ | Every 3 three days from ICU day 2 to 17. | Checked prescription |
| **Patients under adequate sedation**  Patient-days under moderate, mild sedation, or alert and calm (RASS -3 to 0) | $=\frac{number of patient-days under adequate sedation}{patient-days on MV}$ | Every 3 three days from ICU day 2 to 17. | We assessed RASS of patients on invasive mechanical ventilation |
| **Rate of analgesic use** | $= \frac{number of patient-days with analgesic prescription}{patient-days} X 100$ | Every 3 three days from ICU day 2 to 17. | Checked prescription |
| **Tidal volume ≤ 8mL/kg of predicted weight** | $= \frac{patient-days TV\leq8mL per Kg predicted weight}{patient-days on MV} \times1000$ | Every 3 three days from ICU day 2 to 17. | Checked mechanical ventilator data |
| **Central line-associated bloodstream infection (CLABSI) rate** | $= \frac{number of CLABSI}{number of patient-days with central line} \times1000$  *Bloodstream infection defined according to the CDC criteria* | Daily from ICU day 2 to 17. | Blood cultures and clinical data |
| **Rate of ventilator-associated pneumonia (VAP)** | $= \frac{number of PAVs}{number of patient-days on MV} \times1000$ | Daily from ICU day 2 to 17. | Laboratory and clinical data |
| **Rate of urinary tract infection associated with catheter (UTI)** | $= \frac{number of UTIs}{number of patient-days with indwelling urinary catheter} \times1000$ | Daily from ICU day 2 to 17. | Urine culture and clinical data |
| **Rate of mechanical ventilation** | $= \frac{number of patient-days on MV}{number of patient-days} \times100$ | Daily from ICU day 2 to 17. | Patients’ inspection |
| **Rate of use of central line catheter** | $= \frac{number of CVC-day}{number of patient-days} \times100$ | Daily from ICU day 2 to 17. | Patients’ inspection |
| **Rate of use of indwelling urinary catheter** | $=\frac{number of IUC-day}{number of patient-days} x 100$ | Daily from ICU day 2 to 17. | Patients’ inspection |
