# Supplementary material for: A cluster randomized trial of a multifaceted quality improvement intervention in Brazilian intensive care units: study protocol
Source: Implement Sci. 2015 Jan 13;10:8. doi: 10.1186/s13012-014-0190-0 (PMC4342101; doi:10.1186/s13012-014-0190-0)
Supplement: Additional file 3: — Outcomes. Outcomes reflecting care processes. [file 13012_2014_190_MOESM3_ESM.docx]

**Additional file 3** Clinical outcomes

| **Outcome** | **Definition** | **When we assessed** | **How we assessed** |
| --- | --- | --- | --- |
| **In-hospital mortality*** | $= \frac{in-hosptal deaths}{hospital discharges}$ | Hospital discharge. Follow-up limited to 60 days after ICU admission. | We considered only patients eligible for the study |
| **ICU mortality*** | $= \frac{ICU deaths}{ICU discharges}$ | ICU discharge | We considered only patients eligible for the study |
| **Length of ICU stay*** | = Length of ICU (days) | ICU discharge | We considered only patients eligible for the study |
| **Length of hospital stay*** | = Length of hospital (days) | Hospital discharge. Follow-up limited to 60 days after ICU admission | We considered only patients eligible for the study |
| **Mechanical ventilation-free days at 28 days** | = Survival time free of invasive mechanical ventilation in the first 28 days after join the study  Patients who were discharged from hospital alive before 28 days are considered to be alive and free of mechanical ventilation until the 28th day. | 28 days after ICU admission | We considered only patients eligible for the study |

Follow-up is limited to 60 days after ICU admission. Patients with length of hospital stay longer than 60 days will be considered as alive for mortality outcomes and discharged from ICU and hospital.
